# Ultralow-energy amorphization of contaminated silicon samples investigated by molecular dynamics

**DOI:** 10.3762/bjnano.14.68

**Published:** 2023-08-01

**Authors:** Grégoire R N Defoort-Levkov, Alan Bahm, Patrick Philipp

**Affiliations:** 1 Advanced Instrumentation for Nano-Analytics (AINA), Materials Research and Technology Department (MRT), Luxembourg Institute of Science and Technology (LIST), 4422 Belvaux, Luxembourg; 2 University of Luxembourg, 4365 Esch-sur-Alzette, Luxembourg; 3 Thermo Fisher Scientific, Hillsboro, OR, 97124, USA

**Keywords:** angle dependency, argon, contamination, energy dependency, ion bombardment, low energy, molecular dynamics, silicon, simulations, water

## Abstract

Ion beam processes related to focused ion beam milling, surface patterning, and secondary ion mass spectrometry require precision and control. Quality and cleanliness of the sample are also crucial factors. Furthermore, several domains of nanotechnology and industry use nanoscaled samples that need to be controlled to an extreme level of precision. To reduce the irradiation-induced damage and to limit the interactions of the ions with the sample, low-energy ion beams are used because of their low implantation depths. Yet, low-energy ion beams come with a variety of challenges. When such low energies are used, the residual gas molecules in the instrument chamber can adsorb on the sample surface and impact the ion beam processes. In this paper we pursue an investigation on the effects of the most common contaminant, water, sputtered by ultralow-energy ion beams, ranging from 50 to 500 eV and covering the full range of incidence angles, using molecular dynamics simulations with the ReaxFF potential. We show that the expected sputtering yield trends are maintained down to the lowest sputtering yields. A region of interest with low damage is obtained for incidence angles around 60° to 75°. We also demonstrate that higher energies induce a larger removal of the water contaminant and, at the same time, induce an increased amorphization, which leads to a trade-off between sample cleanliness and damage.

## Introduction

Low-energy ion beams offer substantial improvements and possibilities to reduce the damage production on the surface of samples [1–2]. In recent years, the need to control what happens at the surface of the sample has risen sharply, specifically for semiconductors [3–4], microelectronics [5], and surface patterning [6–7]. Other applications of low-energy beams include the preparation of nanoholes [8–9]. Furthermore, deposition processes are substantially more controllable at low energies [10–11], which also makes such beams valid candidates for these processes.

Another application includes lamella preparation for transmission electron microscopy (TEM). TEM and scanning electron microscopy (SEM) have high constraints regarding cleanliness [12]. TEM samples require a small thickness [13] to be electron transparent and are usually prepared through ion milling processes [14–15], which rely heavily on the precise ablation of materials to preserve the crystalline structure of the analyzed sample. These samples, usually shaped as lamellas, are very sensitive to contaminations [16] and environmental changes during preparation to the point where an oxide layer formed on the surface of the lamella could complicate analysis [17–18]. While this oxide layer has a substantial impact, ion-induced damage contributes even more to the degradation of the sample [19] because of in-depth amorphization.

Low-energy ion beams (i.e., impact energies below 1 keV) could offer increased precision during milling [20] as well as substantially reduced damage near the surface of the samples. This de facto preserves the structure of the sample as closely as possible, which includes a minimization of the thickness of the amorphous layer. Focused ion beams of such low energy are generally hard to achieve due to the difficulty of focusing. Some current instruments can utilize focused beams of 500 eV to perform chemical analysis of materials [21], and it is planned to reach down to 50 eV, which would be correspondingly surface sensitive, in the near future.

In the two above cases, contaminations in the experimental chamber play an important role during the sputtering processes. Typical contaminations are (in the order of frequency) water, nitrogen, carbon and carbonated components that can be found in the atmosphere [22–24], and residuals from past experiments in the chamber, which can include silicon, carbon, or any type of particles that were sputtered previously and adsorbed on the walls of the sample chamber [24–25]. The work in this paper will be based on methodologies developed in a previous paper [26], which focused on a silicon sample contaminated with a water layer, and in which we showed the influence of the contamination layer on the sputtering process. In the presence of water on the sample surface, we showed that while the amorphization depth was substantially increased, there was no significant impact on the silicon sputtering yield. In this article, we will consider incidence angles of 0°, 30°, 45°, 60°, 75°, and 83°, defined with respect to the surface normal, and ultralow impact energies of 50, 100, 200, 300, 400, and 500 eV. Our aim is to determine the optimal conditions to reduce damage formation near the surface while also retaining a high degree of contaminant removal. All bombardments have been simulated using a reactive force field (ReaxFF) [27–29] and the molecular dynamics (MD) code LAMMPS [30–31]. While low-energy ion beams are hard to obtain experimentally, such beams are easy to model in simulations. In this paper, by studying the differences between higher (500 eV) and lower (50 eV) impact energies, we will show the differences in the amorphization processes. As expected, higher energies increase amorphization. We will also show that favorable angles to minimize the implantation of the species of the contamination layer are in the range of 60° to 75°.

## Computational Methods

### Force fields

The force fields used to simulate the Ar bombardment of a contaminated silicon sample have already been described in a previous article [26]. To summarize, they are composed of a set of two interatomic potentials. One is the reactive force field (ReaxFF) [29] designed to compute the bonds between silicon, oxygen, and hydrogen atoms, as well as to compute the distribution of partial charges to model the formation and breaking of bonds in the sample. The second one is the Morse potential [32] used to model the interactions of the argon particles with other species. Since argon atoms interact only very weakly with the sample atoms, a simple Morse potential is enough to describe these interactions.

ReaxFF potentials are derived from quantum mechanics calculations [33–34] and allow one to model the formation and breaking of bonds with good precision and reasonable computation costs. For our simulations, this information is of critical importance since it allows us to describe precisely the interactions taking place during the Ar ion bombardment near the sample surface. Oxygen and silicon exhibit a particularly strong interaction [35–36], and partial charges contribute significantly to the bond energy. ReaxFF potentials can describe this phenomenon and allow one to simulate the response of the sample bombarded with different energies and under different angles. The ReaxFF potential uses the QEq charge equilibration method [33,37–38], which is important because this method provides the partial charges for various chemical environments. The force field parameters from the supporting information of [29] were also used in this study.

In contrast, the simplicity of the Morse potential allowed us to represent the interactions between the argons atoms with full valence band and the sample atoms. As described in [26], a set of DFT calculations were performed using VASP to compute the Morse potential for argon–silicon, argon–hydrogen, and argon–oxygen interactions. In the Morse potential, the interatomic interactions depend only on the distance between the two atoms and not on their charge state, which is implicitly taken into account. Hence, the sample is bombarded with neutral atoms, an approach commonly used in MD simulations [39].

### Molecular dynamics setup

The simulation system is made of a box periodic in *X* and *Y* dimensions, and aperiodic in the *Z* dimension. It contains a sample of 5248 atoms (silicon and a thin layer of water molecules). The simulations are performed using the LAMMPS molecular dynamics code on the HPC cluster of the Luxembourg Institute of Science and Technology. The simulations aim to reproduce a continuous sputtering process in experiments. Argon atoms are accelerated (at various angles and energies) towards the sample surface. The resulting collision cascade is modelled in the sample containing all atoms, exported, and re-used for following Ar impacts. By increasing the number of Ar impacts, a higher fluence is reached, for example, 500 Ar impacts on a surface of 18.9 nm^2^ result in a fluence of 2.6 × 10^15^ atoms/cm^2^. These 500 impacts are distributed randomly over the whole (100) sample surface. The velocity vector of the Ar atoms is always parallel to the [010] direction, and it will vary with respect to the [100] direction. For an optimal visualization of the sample structure, most of the images are taken facing the (110) orientation since the lattice is more easily understood using this angle of observation. During the bombardments, a Langevin thermostat is only applied to a slab at the bottom of the sample, which has a thickness of one unit cell. The remaining sample is in the NVE ensemble. Only during the cooldown phase following the development of the collision cascade, the thermostat is applied to the whole sample to get back to equilibrium and get the sample ready for another bombardment. A more in-depth description of the full preparation of the sample and the workflow for continuous ion Ar bombardment is given in [26].

In the current study we will focus on the impact of energy variations on the sputtering processes, while discussing the angle dependency for some conditions. To perform such analysis, we selected several angles and energies. For the impact energy, we selected 50, 100, 200, 300, 400, and 500 eV to stay in the ultralow-energy domain. For the angles, we selected 0°, 30°, 45°, 60°, 75°, and 83° with respect to the surface normal to cover the full range. We will specifically elaborate the discussion of the grazing incidence angles, which are of interest regarding the applications of ultralow-energy Ar ion beams.

The innovative parts of the simulation process are the presence of a contamination layer on top of the sample and the continuous sputtering process, which can be decomposed in two steps: (1) a bombardment step, where argon ions are shot at a specific angle and a specific energy towards the sample, and (2) a cooldown step, where the target is cooled down until it reaches room temperature again. All simulations are carried out at 300 K to reproduce Ar beam processes at room temperature.

## Results and Discussion

**Figure 1:**
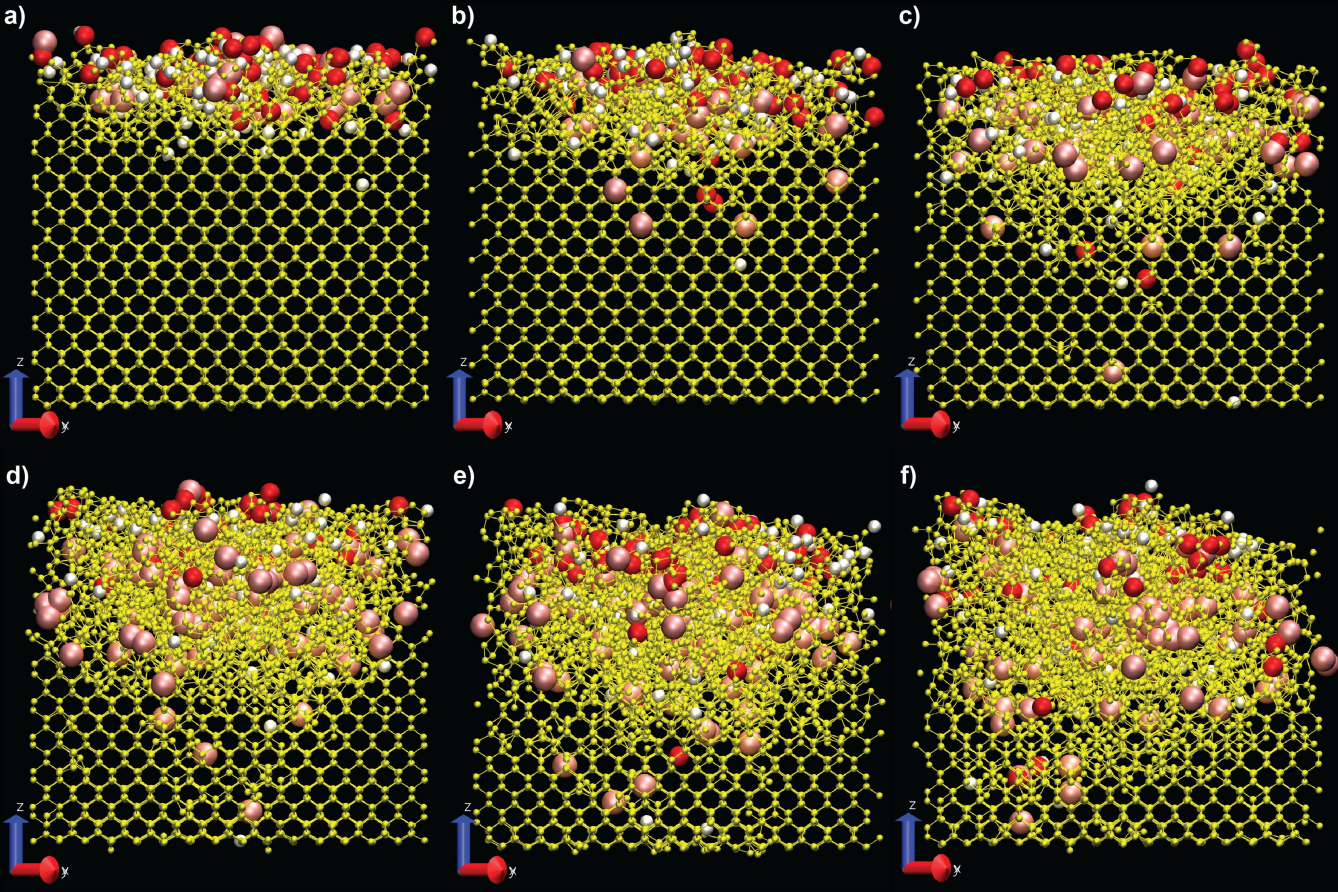
Representations of the contaminated sample under argon irradiation for an incidence angle of 45° and for (a) 50 eV, (b) 100 eV, (c) 200 eV, (d) 300 eV, (e) 400 eV, and (f) 500 eV. The sample is oriented in the (110) direction for visualisation, yet the bombardments are made randomly with the velocity vector parallel to the (010) surface. There are four atom types in the images: The silicon particles are represented in yellow, argon in pink, oxygen in red, and hydrogen in white.

In Figure 1 the status of the sample after 500 impacts (corresponding to a fluence of 2.6 × 10^15^ atoms/cm^2^) is represented for each impact energy investigated in this study and for an incidence angle of 45° with respect to the surface normal and parallel to the (010) surface. We selected 45° for this representation as it is a good angle for the observation of a significant number of Ar implantations (maximal at normal incidence) and for some significant amorphization (maximal at angles of 60–75°). Higher impact energies will induce deeper damage as well as increased sputtering yields, hence the depth of the crater will increase with impact energy.

The methodologies to characterize irradiation-induced sample modifications and sputtering processes are the same as in [26]. In this publication, the focus is on impact energy variations. We should expect significant variations in sputtering yields, damage formation, and amount of material displaced. We will also study fragmentation, implantation, and sputtering of water molecules, which is another interesting aspect of the analysis methodology.

### Amorphization coefficient

The amorphization coefficient used to characterize damage formation in the irradiated sample is a modification of the strain formula adjusted to the needs of the MD simulations in this work. It is described in Equation 1:


[1] $$\mu =\frac{1}{n_{\text{bonds}}}\sum_{n_{\text{bonds}}}\left[1-\left|\frac{\text{bond }{\text{length}}_{\text{theory}}-\text{bond }{\text{length}}_{\text{measured}}}{\text{bond }{\text{length}}_{\text{theory}}}\right|\right].$$

The samples are divided into slabs of equal thickness and of one unit cell height. According to the value of the amorphization coefficient calculated in each slab, we could identify three regions of interest. The first is the crystalline region, which corresponds to values of the amorphization coefficient µ > 0.94 and indicates a pristine crystalline structure. This region is situated closer to the bottom of the sample. The second is the amorphous region, where µ < 0.89 and no local order is present. It is located near the surface. The third region is a transitive region that we label “partially amorphous region”, where 0.89 < µ < 0.94. The damage induced by ion Ar irradiation has not yet completely disturbed the local order. This region is always located between the crystalline and amorphous slabs.

In the crystalline region, the sample is intact, and only thermal vibrations occurs. The amorphous region has been damaged by the recoil atoms and argon, and the crystalline structure is not present anymore. The transitive region is a buffer region in which some damage can be found but the crystalline structure is preserved to some extent. Most of the time, this buffer is one unit cell thick; however, it has higher values in some cases, which will be discussed later. For instance, at normal incidence the damage is localized but penetrates deeply, inducing a thicker transition zone between the crystalline region and the amorphous region. It is important to note how the amorphization takes place. The 500 subsequent bombardments with their collision cascades lead to the displacement of target atoms, creating disorder and point defects. Hydrogen and oxygen atoms get mixed into the target and can get trapped at interstitial or vacancy sites. The different kind of defects are not intrinsically integrated in the amorphization coefficient but get detected by changes in bond lengths that they induce.

**Figure 2:**
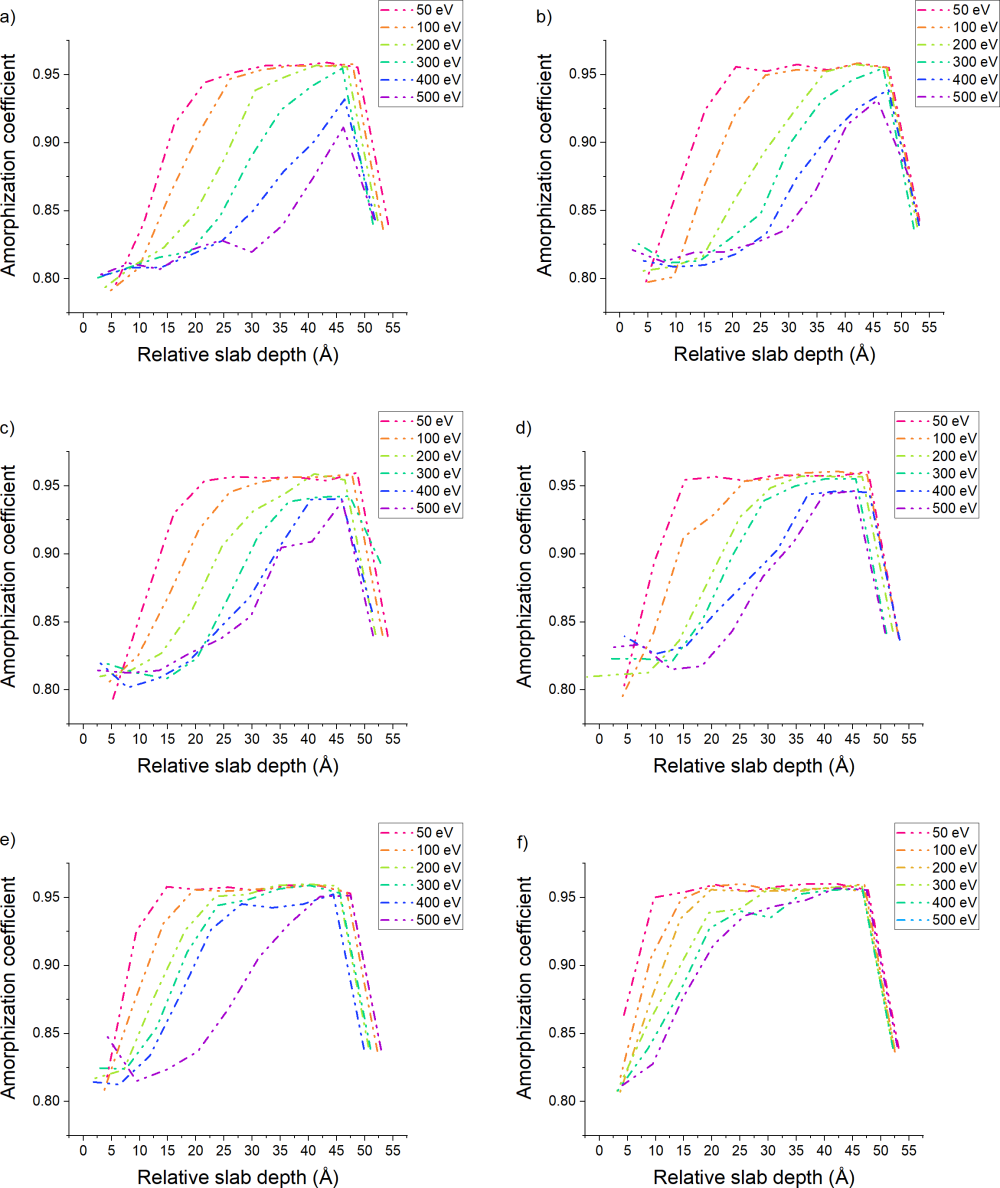
Evolution of the amorphization coefficient as funciton of the energy for (a) 0°, (b) 30°, (c) 45°, (d) 60°, (e) 75°, and (f) 83°. The low values at a depth around 53 Å are due to the dimers formed at the bottom surface of the sample during the equilibration process.

In Figure 2, the variation of the amorphization coefficient is plotted for several angles as a function of the impact energy. For all energies, the thickness of the amorphous region is smaller for higher incidence angles. This can prove especially useful since 500 eV argon beams can be collimated more easily than 50 eV beams; yet, no significant modification of the amorphous layer thickness is observed. To complement the observations on the amorphous layer thickness, we will study in later sections several other parameters to corroborate this hypothesis. Moreover, for 83°, the sample keeps a pristine layer whereas for almost all other angles, at higher impact energies, the sample is damaged to the point where almost no crystalline structure remains (see Supporting Information File 1). Supporting Information File 1 shows the variation of the amorphization coefficient, taking 50 and 500 eV collisions as a basis. We display all angles, allowing for a good comparison of the damage done at higher angles. In this configuration, the partially amorphous slab has an increased thickness because of the implantation of higher-energy argon ions.

**Figure 3:**
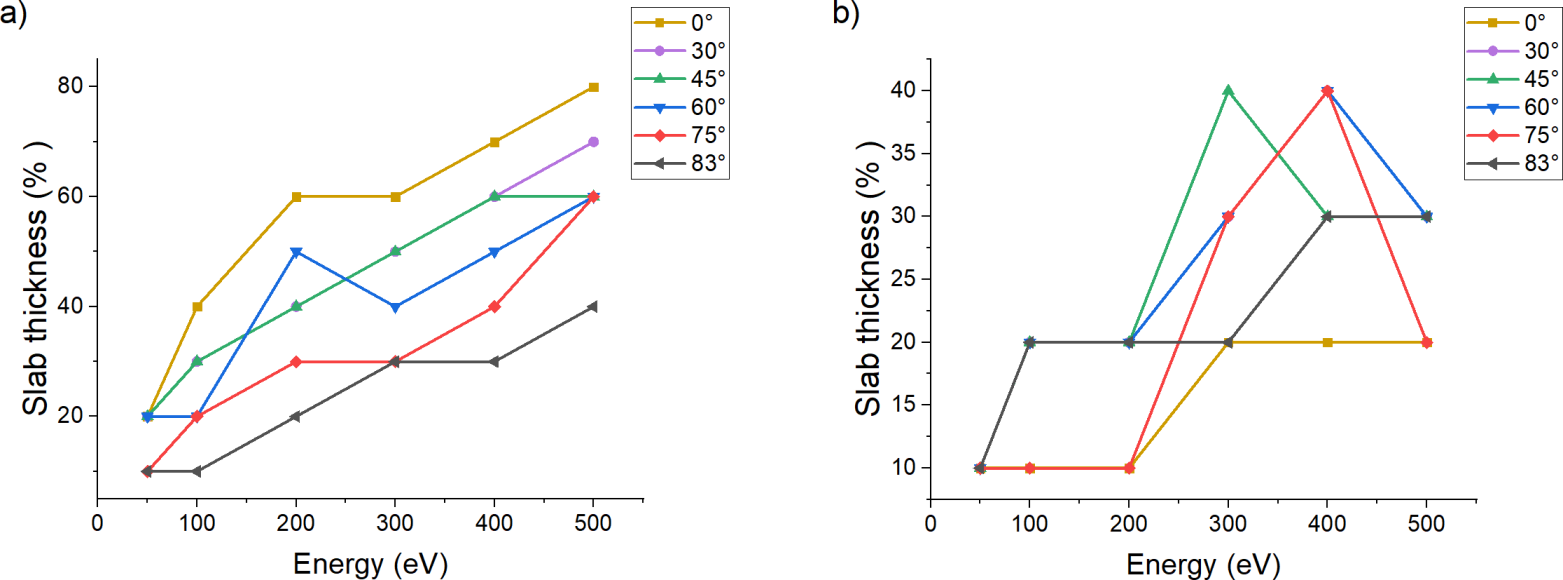
Evolution of the thickness of (a) the amorphous region and (b) the partially amorphous region for each energy with respect to the angle. The *Y* axis measures the thickness in percentage of the initial total sample thickness.

To elaborate the analysis of the amorphization coefficient, we plotted the variation of the thickness for each region of interest for all simulation conditions. In Figure 3, we show the evolution of the slab thickness for the amorphous and partially amorphous regions. The thickness of the crystalline slab is always the difference between the total sample thickness and the thickness of the amorphous and partially amorphous slabs (we included it in Supporting Information File 1). The thickness of the amorphous slab, regardless of the angle, evolves almost linearly with respect to the impact energy. The thickness of the amorphous slab is the same at 83° and 500 eV and at 45° and 200 eV. This supports the idea that grazing incidence collisions cause less damage. The thickness of the partially amorphized region varies due to changes in beam energy and incidence angle and is related to the extent of the collision cascade. This behavior induces a localized concentration of damage which can, at certain angles, channel deeper in the bulk. This, in turn, can cause some extremely localized disorder that would disturb the lattice enough to modify the amorphous coefficient value below the threshold for the crystalline region. An extensive characteristic that can be studied easily in each of our slabs is the radial distribution function, as it allows to confirm the nature of each slab by studying the local order/disorder.

### Radial distribution function

**Figure 4:**
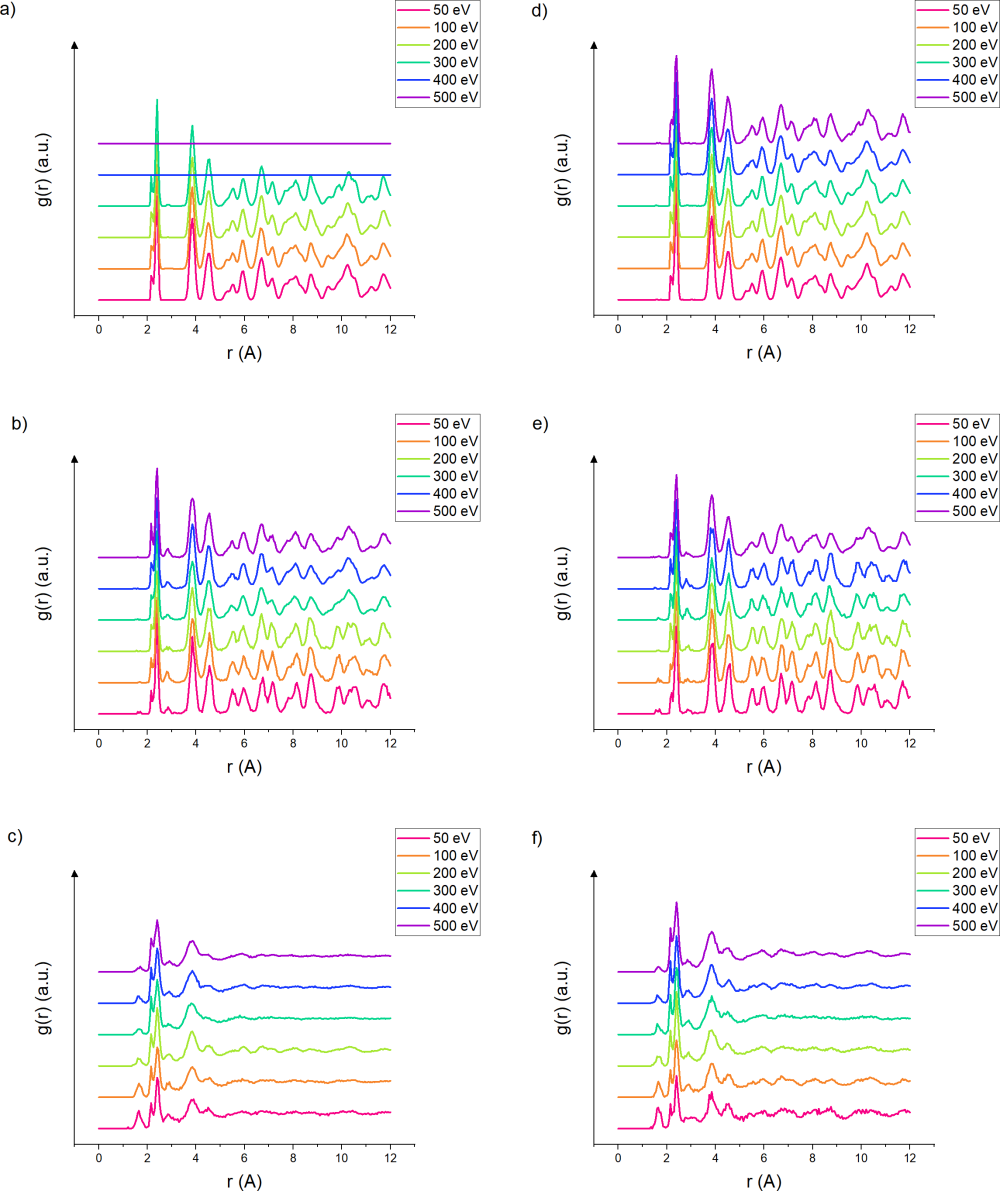
Evolution of the RDF for each slab at collisions under 30° and 75° incidence from crystalline to amorphous for (a–c) 30° and (d–f) 75°. In (a), the continuously straight line indicates a non-existing region, that is, the sample turned completely amorphous or semi-amorphous under ion bombardment. The *Y* axis is normalized to the density and given in a stacked representation. The intensity of the peaks describes the probability to find an atom at a given distance of a reference atom.

The radial distribution function (RDF) allows one to study the variations in crystalline structure in the sample by quantitatively characterizing the number of atomic neighbors. The RDF acts as a good estimator of the local order (or disorder) of the sample and complements the amorphization coefficient. The RDF was computed using the algorithm provided by Kopera et al. [40] for each slab of one unit cell height. Each slab is attributed to one of the three regions (i.e., crystalline, partially amorphous, and completely amorphous) based on its amorphization coefficient calculated in the previous section. The partially amorphous region is the transition zone between the crystalline and the amorphous region. In Figure 4 we can observe the variations of the RDF with respect to the energy for 30° and 75° argon bombardments for each of the three regions. The two angles of 30° and 75° were chosen because they represent the two cases of normal/close-to-normal incidence (30°) and close-to-grazing-incidence angles (75°). For clarity, the other angles have not been included, yet the conclusions are similar to the ones listed in the following paragraph. Between amorphous and crystalline regions, there is a transition zone in which some of the Si atoms get displaced, modifying the local order by broadening the peaks, while the crystalline structure is maintained in other places, keeping a long-range order above 4 Å with the presence of distinct peaks. The graphs in Figure 4b and Figure 4e represent this transition region and are not an overlay of the graphs in Figure 4a and Figure 4c or in Figure 4d and Figure 4f, respectively.

Interesting kinds of behavior can be best observed when the data is sorted with respect to energy. At higher energies (above 400 eV), we observe a complete removal of the crystalline slab, overrun by the thickness of amorphous and transition regions. This is observed in the graphs for the crystalline regions (Figure 4a and Figure 4d) and consistent with the observations made above. There is a correlation between lower impact energy, increased contaminant implantation, and decreased argon implantation. The silicon–oxygen and silicon–hydrogen bond lengths are 1.60 and 1.46 Å, respectively, and give a peak in this region, which can be used to estimate the water implantation. At lower energies, the peak intensities related to water species are at their highest, whereas the argon implantation peak seems to be at its lowest. The behavior is the same at 83°, but it disappears at higher energies. To preserve the readability, only RDF results for 75° are displayed. The RDF graphs for the other angles can be found in Supporting Information File 1. At energies above 400 eV and for angles above 60°, the peak intensities related to water species (i.e., from Si–O and Si–H interactions) are strongly reduced, which indicates a significant removal of the contaminant. This translates to an overall increased sputtering yield as will be discussed in a later section.

To further confirm the hypothesis that higher-energy argon atoms at grazing incidence do not produce amorphization, we will proceed to analyze the implantation depths of the contaminants and argon atoms in the sample.

### Implantation depth

**Figure 5:**
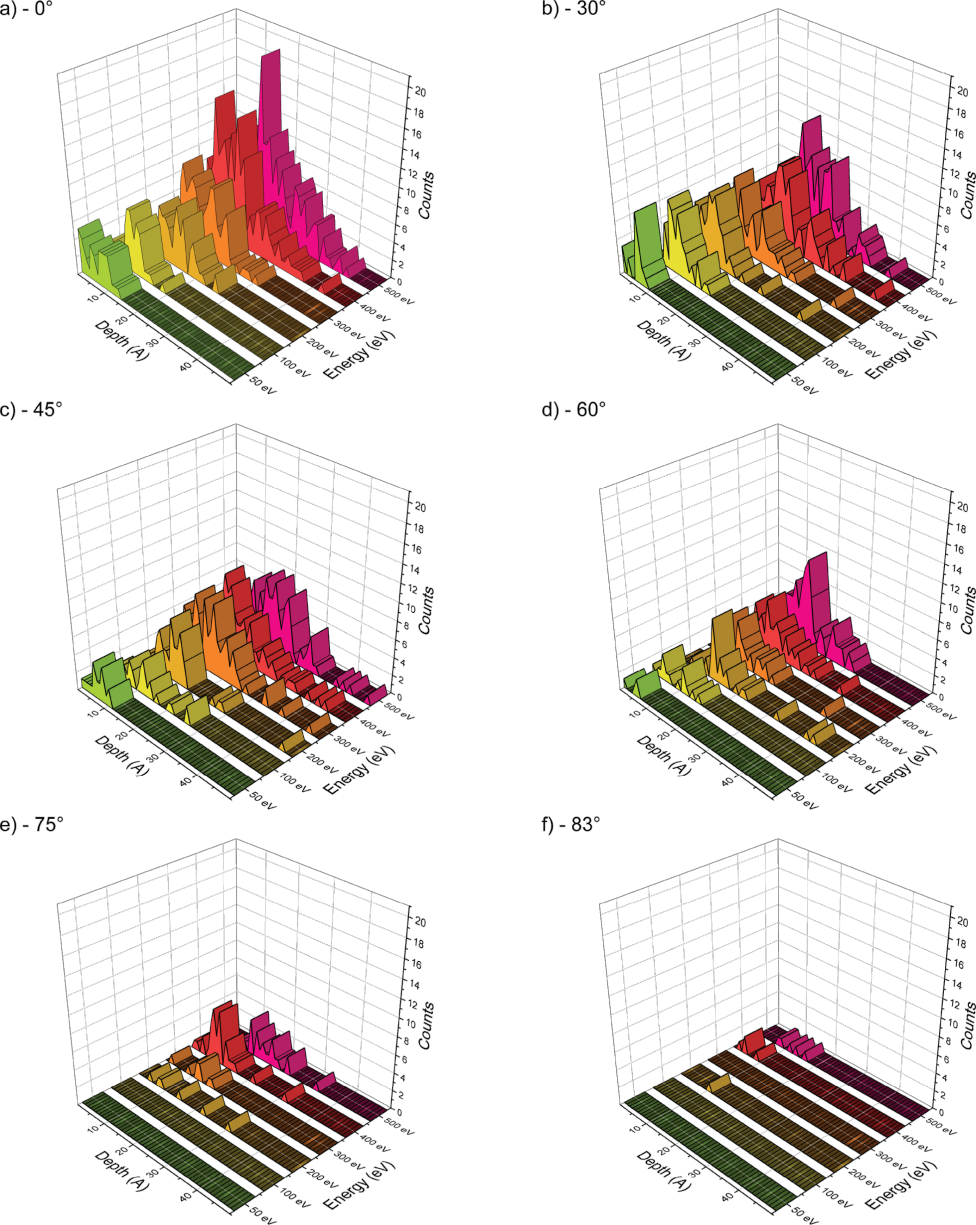
Implantation depth of argon atoms for incidence angles of (a) 0°, (b) 30°, (c) 45°, (d) 60°, (e) 75°, and (f) 83°. The energy is displayed on the *Y* axis, the implantation depth of each implanted particle is measured along the *X* axis, and the counts are displayed in the *Z* axis. The color code is used to differentiate the different energies and is solely for visualization purposes.

**Figure 6:**
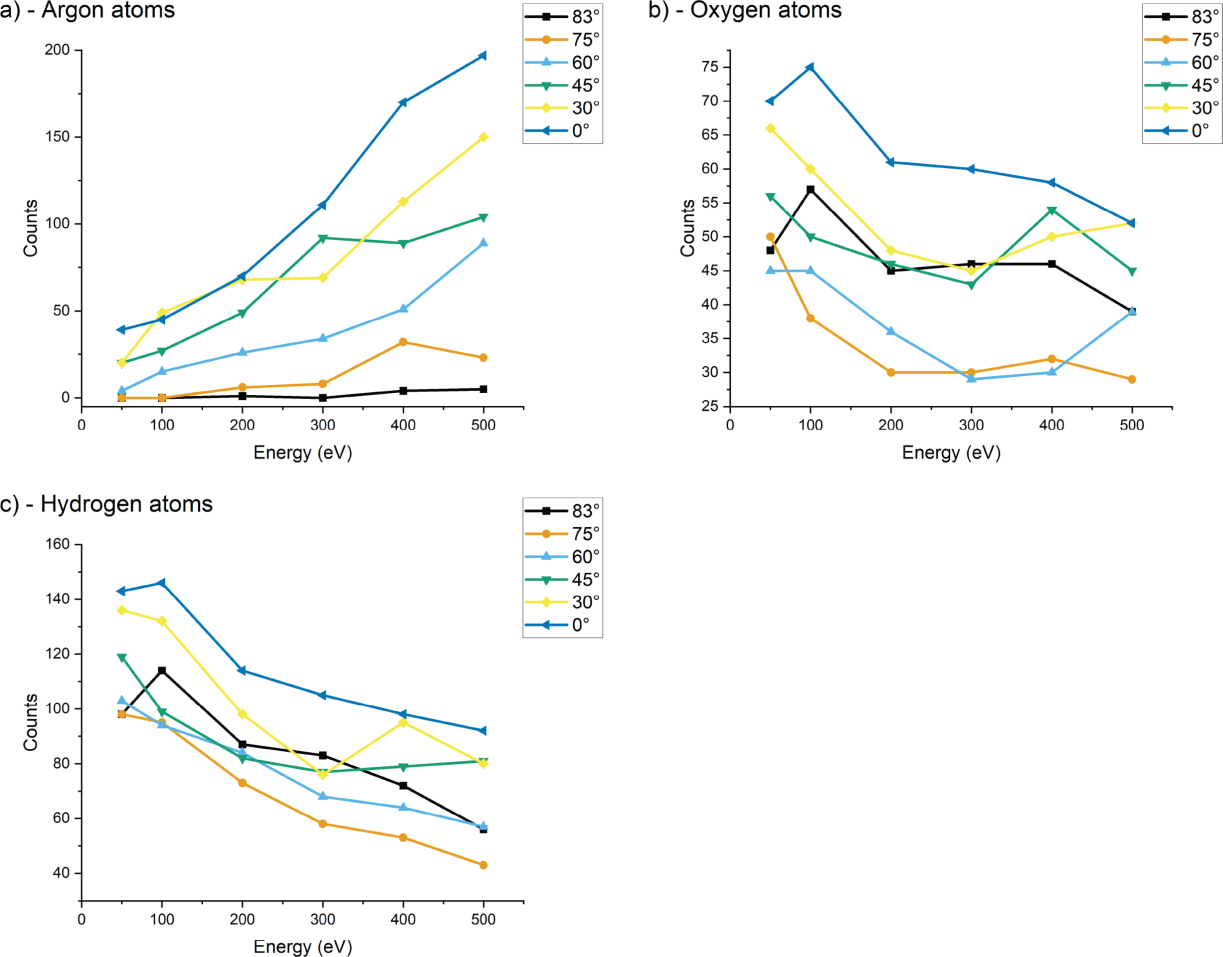
Total implantation counts of (a) argon atoms, (b) oxygen atoms, and (c) hydrogen atoms in the sample with respect to the impact energy for the different incidence angles. Because the irradiation process is limited to 500 bombardments, there are statistical variations between the different conditions leading to some outliers (i.e., 400 eV and 75° compared to 500 eV and 75° for argon). For each species, the counts have been integrated over all slabs, that is, the whole sample depth.

**Figure 7:**
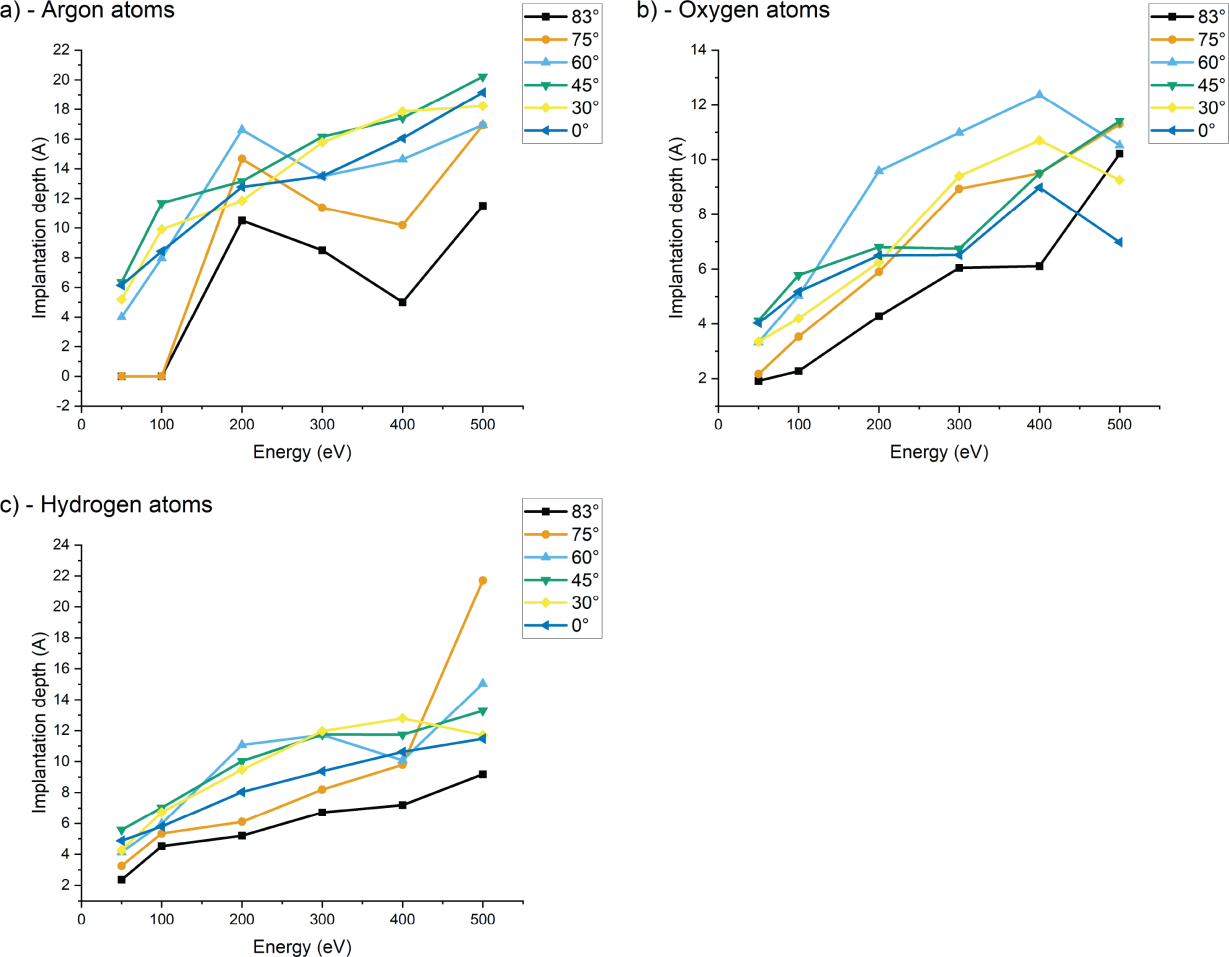
Mean implantation depth of (a) argon atoms, (b) oxygen atoms, and (c) hydrogen atoms in the sample with respect to the impact energy for the different incidence angles. Higher values indicate an increased concentration of the particle type. There are significant statistical variations because the irradiation simulation is limited to 500 impacts.

The distribution of implanted argon is extremely sensitive to the impact energy and the incidence angle, and so is the implantation of the atoms from the water molecules. Their implantation depends on the energy transmitted to the water molecules via elastic collisions with argon atoms or other species in the sample. Figure 5 shows the evolution of the implantation depth of argon atoms for the different experimental conditions. These graphs show the importance of the incidence angle when low implantation depths are required. With increasing energy, the implantation depth is increasing, as expected. The argon implantation depth is minimized at grazing incidence. For angles above 75°, even at higher energies, the simulation results show that almost no argon is implanted into the sample. The same implantation graphs for oxygen and hydrogen are shown in Supporting Information File 1. Figure 6 shows the amount of argon, hydrogen, and oxygen implanted in the sample, and Figure 7 shows the mean implantation depths for the three species. These two figures summarize all implantation distributions.

While we can see in Figure 6 a quasi-linear decrease of the counts of oxygen and hydrogen, we can observe that argon implantation is very sensitive to energy and angle. The implantation is maximal at 500 eV, which was expected. Yet, we see that the highest angles lead to a minimal implantation of argon, while there are low counts for both oxygen and hydrogen for almost all angles. Since the irradiation process is limited to 500 impacts, statistical variations are obtained and lead to outliers in the counts for each particle type. For example, we can observe a discrepancy between 400 and 500 eV for argon at 75°. Similarly, there are some fluctuations in the oxygen data at 400 eV.

Figure 7 shows a linear variation of the implantation depth with energy, which should be expected since higher energies will induce deeper penetration in the sample. For some conditions, the number of implanted species is small, which explains some fluctuations in the data. Overall, more argon particles are implanted and their implantation is deeper. This trend is minimized at 83°, while the removal of contaminant is not minimized at these high angles. The last point will be discussed in the section on the sputtering of clusters.

For 50 eV argon, there is almost no implantation. The impact energy is barely enough for argon to penetrate into the sample, and the argon atoms will remain extremely close to the surface. A lot of backscattering is observed. At 500 eV, the argon implantation is increased; however, at grazing incidence angles the implantation remains minimal. This is interesting for milling processes where the sample should stay as pristine as possible. Since there is almost no argon implantation, it is safe to assume that the contaminant will play a major role in the amorphization of the sample. This observation is especially interesting as we can find some conditions where the contaminants are quite efficiently removed. We began to underline this set of conditions in the previous section, and we will pursue the discussion in the forthcoming sections.

In Figure 7, we can see that the mean implantation depths of the contaminants are increasing with impact energy, as expected, with a slight difference between oxygen and hydrogen, which is related to their mobility. Because of the electronegativity of oxygen and its strong interaction with silicon, we can assume that free oxygen will tend to pair extremely quickly with silicon, whereas hydrogen can travel deeper into the sample, because of its low mass and via channeling processes. Contaminants tend to remain closer to the surface than argon. Because the water molecules are deposited at the surface, even higher energy collisions will only transmit a fraction of the energy to the water molecules, which will be either sputtered or fragmented and implanted. Hence, the resulting penetration depth of hydrogen and oxygen should be shallower than for argon. It is interesting to observe that at higher energies, the total number of implanted oxygen and hydrogen atoms is reduced, meaning that a higher energy will favor the removal of contaminants. This effect is further enhanced at angles above 75°. An angle of 83° and an energy of 500 eV are the most favorable conditions for contaminant removal. Sample purity is also enhanced since fewer argon atoms are implanted into the sample. This hypothesis regarding contamination removal will be confirmed in the section on the sputtering yields.

### Sputtering yield

The sputtering yield in ion irradiation experiments is especially interesting since it is one of a few parameters that can be measured. Hence, sputtering yields have been thoroughly studied in the past; yet, at low impact energies of 500 eV and below, they are less well known. Only few studies investigated ultralow-energy argon impacts [41–47], let alone the variation of the sputtering yields with respect to angle variations. Previous studies differ from ours in several aspects. Zalm et al. [44] have extensively studied the energy dependence. However, the impact angle was kept at normal incidence, and the energy of impact was far greater than the range studied in this publication. Despite these differences, we could compare some of our results for two energies below the 500 eV threshold. More experimental data is provided in a paper from Wittmaack [48]. In the publication by Timonova et al. [43], the MEAM potential is used to model 500 eV impacts at 45°, which corresponds to a specific case of our study and allowed us to cross-check another data point. In the work of Sycheva et al. [47], the ZBL potential is used to examine 50–300 eV impacts at normal incidence, which is similar to our methodology and allowed us to compare our ReaxFF potential results with those from the different potentials they used. In the investigations described by Lee et al. [45], Ar^+^ and O_2_^+^ ions were used to perform the sputtering, and the publication describes extensively the case for 0°, 40°, and 70° impacts at 500 eV. Our methodology involved an in-depth analysis of several angles as well as several energies in the extremely-low-energy range (between 50 and 500 eV). While this methodology is presented here for silicon, there is no doubt it could be applied to other materials. The key aspect, and what makes this publication new in the scope of the previously cited publications, is the range of angles and energies that have been studied, as well as the presence of a contamination layer.

**Figure 8:**
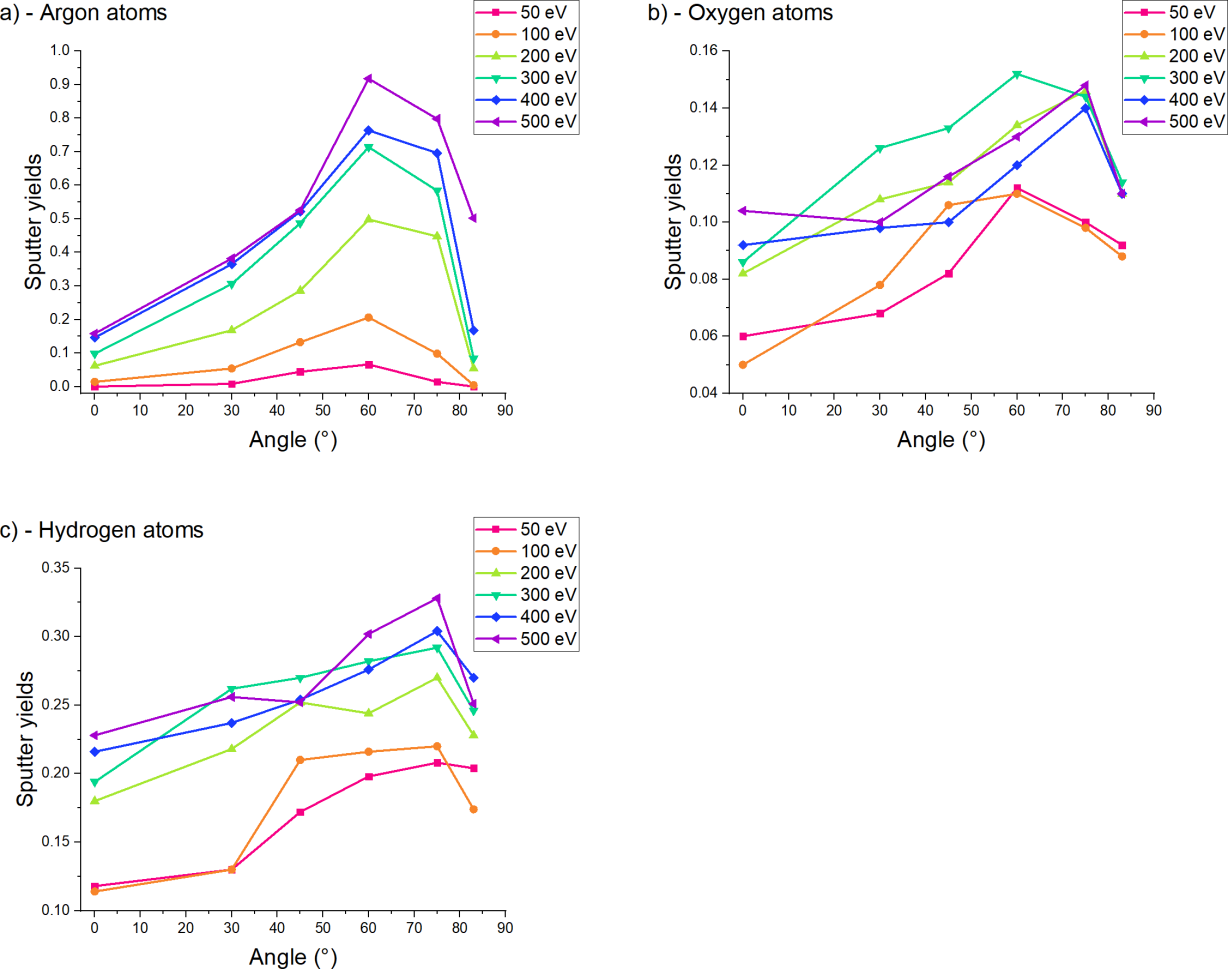
Partial sputtering yields for (a) silicon, (b) oxygen, and (c) hydrogen with respect to the angle for each energy. The energy is displayed with a color code, while the angle is given on the *X* axis, and the yield is displayed along the *Y* axis. In all cases, we observe a maximum at 60° for silicon. The maximum is slightly shifted around 70° for oxygen and hydrogen.

**Figure 9:**
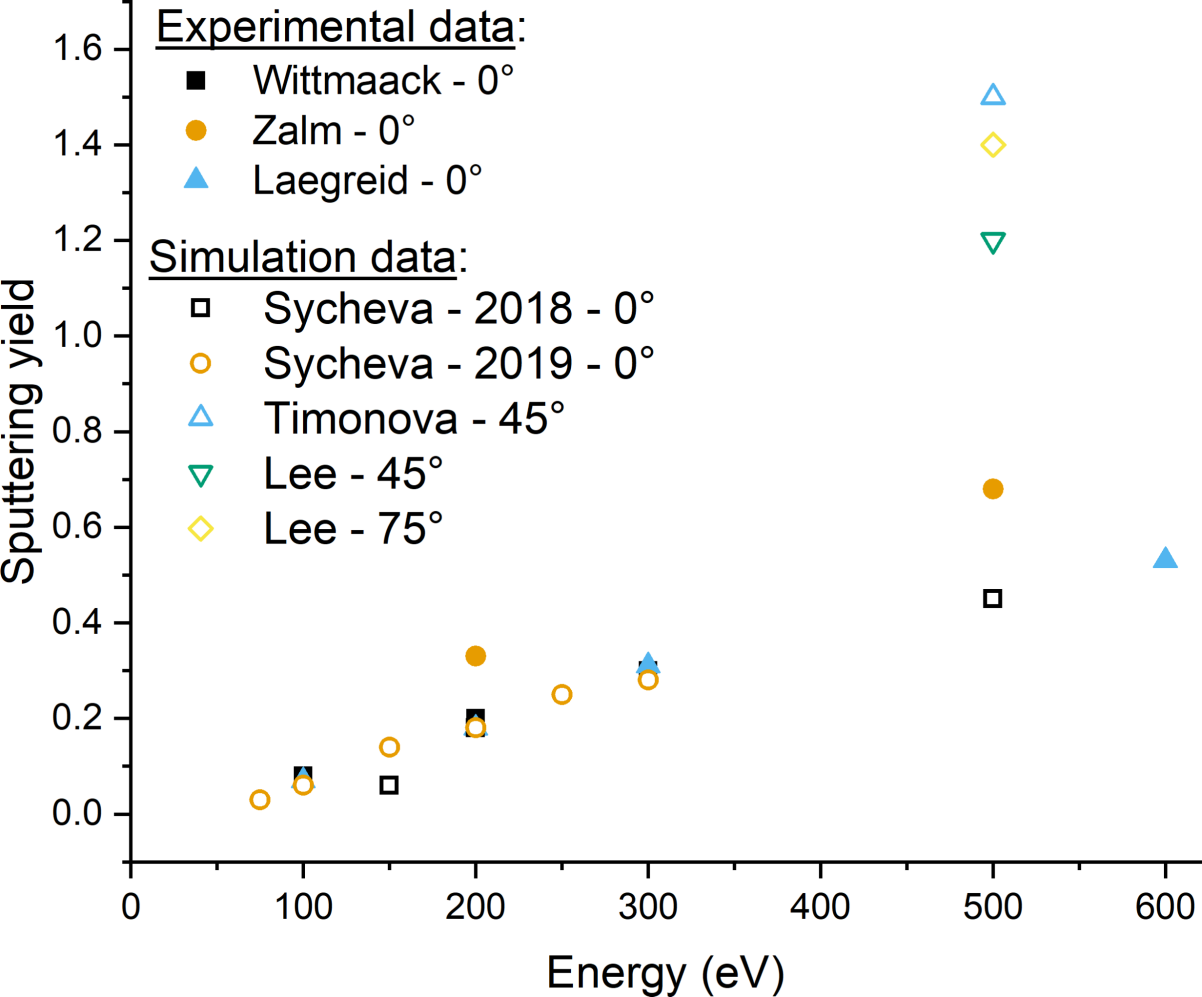
Comparison between experimental data and simulation data found in the literature. Full symbols represent experimental data, while open symbols represent simulation data. The data is taken from [42–45,47–48].

We have plotted the sputtering yields for all impact energies and incidence angles to try to understand the mechanisms of sputtering at such low energies and compared the contaminated to the pristine sample (Figure 8). The silicon sputtering yields follow the expected trend with a maximum yield obtained for an incidence angle of 60°. In Figure 8a, we can observe the increasing impact of the contaminant. For low impact energies (50–100 eV), the contaminant has a minimal impact on the sputtering yield (>5%). However, when increasing the energy, we can observe an increased effect of the contaminant on the sputtering yield, leading to lower silicon sputtering yields. This effect is maximal at 500 eV, where we can see a difference of approximatively 20%. This can be explained by the fact that the low yield variations of low-energy sputtering events (below 100 eV) can be encompassed in statistical variations and are less easy to detect than the yields of high-energy sputtering events (300–500 eV). At higher energies, we can observe that the contaminant forms a shielding layer on top of the sample, which will be preferentially sputtered, as we will see in the next paragraphs. For impact energies below 100 eV, the sputtering threshold is barely reached, and the sputtering yield is minimal. At higher energies, the sputtering yields of our simulations are comparable to the data from literature when identical conditions are available. A comparison of the sputtering yields is shown in Figure 9. At 100 eV and normal incidence, reference values change from 0.06 to 0.08. Our values of 0.03 for the clean sample is lower, which could be due to the low number of events. The contamination lowers the sputtering yield to 0.014. At 200 eV and normal incidence, the reference values range from 0.18 to 0.33. Our sputtering yield of the clean sample of 0.12 is already closer to that range. The contamination lowers the sputtering yield by a factor of 2. At 500 eV and normal incidence, reference values change from 0.45 to 0.68, which agrees well with our value of 0.66 for the clean sample. At this energy, the contamination has only a minor effect on the sputtering yield. Our value of 0.53 is in the range reported in literature. For non-grazing incidence, the values by Sycheva et al. agree well with our values, while the other values are well above. The difference in sputtering yields between our results and the literature data can be explained by the difference between the ReaxFF potential used in our simulations and the force fields or experimental methods used in the other articles. Despite showing slight variations in values, the trends are almost identical. Unfortunately, none of the articles studied the effect of the incidence angle. Hence, this trend could not be compared.

Regarding a minimal sample modification under ion beam irradiation, higher energies increasing the sputtering yield. At the same time, the damage done to the sample due to increased implantation depths must be avoided. A contamination layer should be removed but not implanted. When analyzing the sputtering yields of contaminants, we observe the same trend as for silicon. Higher energies increase the yield, and we can still observe some angle dependency, with a maximum sputtering yield for angles between 60° and 75°. A difference appears above 80°, where the sharp decrease observed for silicon particles is less pronounced for water. In our simulations, the water layer is not renewed between each collision; therefore, the sputtering yields of the water-related species tend to decrease with increasing fluence, that is, they do not follow the same trend as those of silicon. Yet, we can still observe that higher energies tend to give an increased sputter yields for both oxygen and hydrogen particles. Since higher energies also increase the contaminant removal, the trade-off between the removal of contaminant and the increased amorphization depth is relevant for experimental setups in which the sample has been exposed to air for extensive periods of time or for experimental chambers with low vacuum levels. Furthermore, at 83° we can observe a notable drop in silicon sputtering yields, while the sputtering yields for O and H remain high, which makes these conditions favorable for the removal of contaminations.

Combined with the previous observations of implantations and damage formation, we see a trend where 500 eV collisions around 60° incidence would be most suitable to mill silicon samples with high speed. When the minimization of the amorphous layer is important, the impact energy should be lowered. When approaching higher grazing incidence angles than those studied in this work (e.g., to 87°), lower sputtering yields and a thinner amorphous layer can be expected. Figure 6 shows that the number of implanted hydrogen and oxygen atoms is smallest at 75°. Hence, contaminations are removed best at this angle, as can also be seen in Figure 8b and Figure 8c. In an example process, contaminations could be removed first by starting the milling around 75° and changing towards higher angles after some time to minimize the thickness of the amorphous layer. The impact energy can also be lowered down to 50 eV to minimize amorphization further. The latter is of interest, for example, for the preparation of TEM samples.

**Figure 10:**
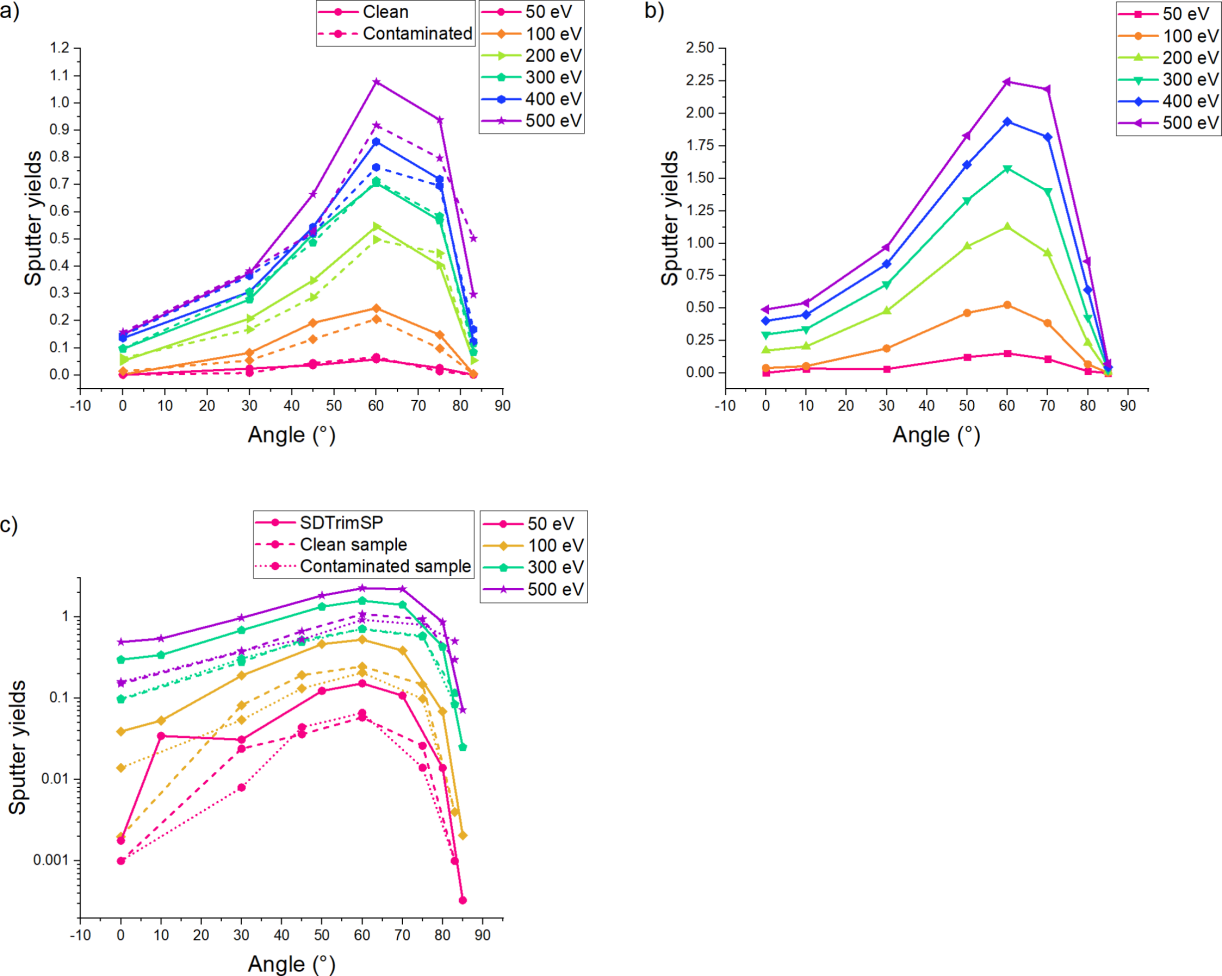
Sputtering yields for (a) MD simulations of pristine and contaminated silicon and (b) SDTrimSP simulation of a clean silicon sample. (c) Logarithmic plot comparing the sputter yields from (a) and (b).

Furthermore, we performed binary collision approximation (BCA) simulations on pristine silicon using the SDTrimSP code [49]. In Figure 10, the sputtering yields are plotted, as well as a comparison between the three sputtering simulations of the silicon sample. We can observe a very similar trend for MD and SDTrimSP, yet we also observe a significant difference between the yields (Figure 9). This can be explained by the difference in the codes used. While ReaxFF models breaking and forming of bonds between atoms, the SDTrimSP BCA-type simulations are used as a reference method for comparison. Furthermore, SDTrimSP and TRIM both use a fully amorphous target and use purely repulsive interatomic potentials. When comparing closely the results for ReaxFF, SDTrimSP, and TRIM, we observe that the maximum yield is obtained around 60°–70° for all models.

More detailed information on the sputtering processes, and in particular on the sputtering of clusters and on the evolution of the fraction of intact water molecules can be found in Supporting Information File 1.

## Conclusion

From all the observations made before we can distinguish several interesting cases for the sputtering of silicon covered with a water layer by ultralow-energy argon ions. For applications where the lowest amount of sample damage is required, higher impact energies need to be avoided since the thickness of the amorphous layer increases with energy. For such applications, grazing incidence angles and energies down to 50 eV should be used. For higher impact energies, we show that the sputtering yields of water molecules and fragments are increased and maximized around 75°, leading to low concentrations of contaminating species in the sample. Hence, such conditions can be used when the removal of surface contaminations is most important. For these conditions, a competition between (1) the amorphization induced by the collisions, by the water fragments, and by the implantation of contaminant species, and (2) the reduced implantation of contaminants due to higher sputtering yields is observed. We observe that grazing incidence collisions minimize the thickness of the amorphous layer while also maintaining decent sputtering yields for contaminating particles. More specifically, at 83° incidence, regardless of the energy of impact, we observe that the amorphous layer thickness is significantly reduced compared to other angles. Depending on the requirements, impact energy and incidence angle can be changed during the process to favor milling, contamination removal, or minimal sample amorphization. These conditions are of interest for applications such as TEM lamella preparation or milling processes.

Simulations give us access to atomic-scale parameters that cannot be obtained in experiments and which are interesting for the fundamental understanding of sputtering mechanics. These simulations were performed with only one kind of contaminant, namely water, and without renewal of the water layer. Future simulations could include other types of contaminants, such as nitrogen or carbon-containing impurities that are very often found in instrument chambers, and the renewal of the contamination layer during the sputtering process.

## Supporting Information

File 1 Additional simulation data.

## References

[1] Mach J, Šamořil T, Voborný S, Kolíbal M, Zlámal J, Spousta J, Dittrichová L, Šikola T (2011). Rev Sci Instrum.

[2] Nebiker P W, Döbeli M, Mühle R, Suter M, Vetterli D (1996). Nucl Instrum Methods Phys Res, Sect B.

[3] Moreno-Barrado A, Castro M, Gago R, Vázquez L, Muñoz-García J, Redondo-Cubero A, Galiana B, Ballesteros C, Cuerno R (2015). Phys Rev B.

[4] Hecht J-D, Frost F, Hirsch D, Neumann H, Schindler A, Preobrajenski A B, Chassé T (2001). J Appl Phys.

[5] Gotoh Y, Kagamimori K, Tsuji H, Ishikawa J (2002). Surf Coat Technol.

[6] Teichmann M, Lorbeer J, Ziberi B, Frost F, Rauschenbach B (2013). New J Phys.

[7] Gago R, Vázquez L, Cuerno R, Varela M, Ballesteros C, Albella J M (2002). Nanotechnology.

[8] Fritzsche M, Muecklich A, Facsko S (2012). Appl Phys Lett.

[9] Wei Q, Zhou X, Joshi B, Chen Y, Li K-D, Wei Q, Sun K, Wang L (2009). Adv Mater (Weinheim, Ger).

[10] Ensinger W (1997). Nucl Instrum Methods Phys Res, Sect B.

[11] Amano J, Bryce P, Lawson R P W (1976). J Vac Sci Technol (N Y, NY, U S).

[12] Soong C, Woo P, Hoyle D (2012). Microsc Today.

[13] Zhang G P, Schwaiger R, Volkert C A, Kraft O (2003). Philos Mag Lett.

[14] Rubanov S, Munroe P R (2004). Micron.

[15] Giannuzzi L A, Drown J L, Brown S R, Irwin R B, Stevie F A (1998). Microsc Res Tech.

[16] Bender H, Franquet A, Drijbooms C, Parmentier B, Clarysse T, Vandervorst W, Kwakman L (2015). Semicond Sci Technol.

[17] Grant J T, Walck S D, Scheltens F J, Voevodin A A (1997). MRS Online Proc Libr.

[18] Rodenburg C, Jepson M A E, Bosch E G T, Dapor M (2010). Ultramicroscopy.

[19] Lotnyk A, Poppitz D, Ross U, Gerlach J W, Frost F, Bernütz S, Thelander E, Rauschenbach B (2015). Microelectron Reliab.

[20] Mehrtens T, Bley S, Venkata Satyam P, Rosenauer A (2012). Micron.

[21] Gazzadi G C, Mulders J J L, Trompenaars P, Ghirri A, Rota A, Affronte M, Frabboni S (2011). Microelectron Eng.

[22] Roediger P, Wanzenboeck H D, Hochleitner G, Bertagnolli E (2009). J Vac Sci Technol, B: Microelectron Nanometer Struct–Process, Meas, Phenom.

[23] Abdel-Samad S, Abdel-Bary M, Kilian K (2005). Vacuum.

[24] Ren Y, Lei D, Yao F, Wang Z (2019). AIP Conf Proc.

[25] Pal G, Yadav R C, Akhter J, Das T, Sarkar A, Mallik C, Bhandari R K (2012). J Phys: Conf Ser.

[26] Defoort-Levkov G R N, Bahm A, Philipp P (2022). Beilstein J Nanotechnol.

[27] van Duin A C T, Dasgupta S, Lorant F, Goddard W A (2001). J Phys Chem A.

[28] Fogarty J C, Aktulga H M, Grama A Y, van Duin A C T, Pandit S A (2010). J Chem Phys.

[29] Newsome D A, Sengupta D, Foroutan H, Russo M F, van Duin A C T (2012). J Phys Chem C.

[30] Plimpton S (1995). J Comput Phys.

[31] Thompson A P, Aktulga H M, Berger R, Bolintineanu D S, Brown W M, Crozier P S, in 't Veld P J, Kohlmeyer A, Moore S G, Nguyen T D (2022). Comput Phys Commun.

[32] Costa Filho R N, Alencar G, Skagerstam B-S, Andrade J S (2013). EPL.

[33] Aktulga H M, Fogarty J C, Pandit S A, Grama A Y (2012). Parallel Comput.

[34] Cowen B J, El-Genk M S (2016). Comput Mater Sci.

[35] Huang L, Kieffer J (2003). J Chem Phys.

[36] Kulkarni A D, Truhlar D G, Goverapet Srinivasan S, van Duin A C T, Norman P, Schwartzentruber T E (2013). J Phys Chem C.

[37] Rappe A K, Goddard W A (1991). J Phys Chem.

[38] Nakano A (1997). Comput Phys Commun.

[39] Smith R (1997). Atomic and Ion Collisions in Solids and at Surfaces.

[40] Kopera B A F, Retsch M (2018). Anal Chem (Washington, DC, U S).

[41] Hofsäss H, Stegmaier A (2022). Nucl Instrum Methods Phys Res, Sect B.

[42] Sycheva A A, Voronina E N, Rakhimova T V (2018). J Surf Invest: X-Ray, Synchrotron Neutron Tech.

[43] Timonova M, Lee B-J, Thijsse B J (2007). Nucl Instrum Methods Phys Res, Sect B.

[44] Zalm P C (1983). J Appl Phys.

[45] Lee H-I, Moon D W, Shin H C, Oh S K, Kang H J (2004). Nucl Instrum Methods Phys Res, Sect B.

[46] Shumilov A S, Amirov I I (2020). J Surf Invest: X-Ray, Synchrotron Neutron Tech.

[47] Sycheva A A, Voronina E N, Palov A P (2019). J Surf Invest: X-Ray, Synchrotron Neutron Tech.

[48] Wittmaack K (2003). Phys Rev B.

[49] Mutzke A, Schneider R, Eckstein W, Dohmen R, Schmid K, von Toussaint U, Bandelow G (2019). SDTrimSP Version 6.00, IPP Report 2019-02.

