# Supplementary material for: Ultralow-energy amorphization of contaminated silicon samples investigated by molecular dynamics
Source: Beilstein J Nanotechnol. 2023 Aug 1;14:834–49. doi: 10.3762/bjnano.14.68 (PMC10407782; doi:10.3762/bjnano.14.68)
Supplement: File 1 — Additional simulation data. [file Beilstein_J_Nanotechnol-14-834-s001.pdf]

BEILSTEIN JOURNAL OF NANOTECHNOLOGY

# Supporting Information

for

# Ultralow-energy amorphization of contaminated silicon samples investigated by molecular dynamics

Grégoire R. N. Defoort-Levkov, Alan Bahm and Patrick Philipp

*Beilstein J. Nanotechnol.* **2023,** *14,* 834–849. doi:10.3762/bjnano.14.68

# Additional simulation data

License and Terms: This is a supporting information file under the terms of the Creative Commons Attribution License (https://creativecommons.org/licenses/by/4.0). Please note that the reuse, redistribution and reproduction in particular requires that the author(s) and source are credited and that individual graphics may be subject to special legal provisions.
The license is subject to the *Beilstein Journal of Nanotechnology* terms and conditions: (https://www.beilstein-journals.org/bjnano/terms)

# Index

## Publication supplements

**Figure S1:** Evolution of the amorphization coefficient for a specific case comparing 50 and 500 eV for each angle.

**Figure S2:** Evolution of the thickness of the crystalline slab with respect to the energy of impact and the angle.

**Figure S3:** Implantation depth charts for each angle and energy for oxygen atoms, full display of the distributions.

**Figure S4:** Implantation depth charts for each angle and energy for hydrogen atoms, full display of the distributions.

## Sputtering of clusters

**Figure S5:** Partial sputter yields for clusters with minimal sputter yields (Si – Si, Si – O, Si – H and entire water molecules).

**Figure S6:** Partial sputter yields for clusters with minimal sputter yields (O – O, H – H and O – H).

## Evolution of the fraction of intact water molecules

**Figure S7:** Probability to fragmentation a water molecule per impact with respect to the fluence for each energy at 0°, 30°, 45°, 60°, 75°, 83°.

**Figure S8:** Probability to fragment a water molecule per impact with respect to the fluence for each angle for a) 50 eV impacts and b) 500 eV impacts.

## ReaxFF Potential

**Table S1:** Force field parameters, taken from [1].

## References

(1) Newsome, D. A.; Sengupta, D.; Foroutan, H.; Russo, M. F.; van Duin, A. C. T. *J. Phys. Chem. C* **2012**, *116*, 16111–16121. doi:10.1021/jp306391p

## Publication supplements

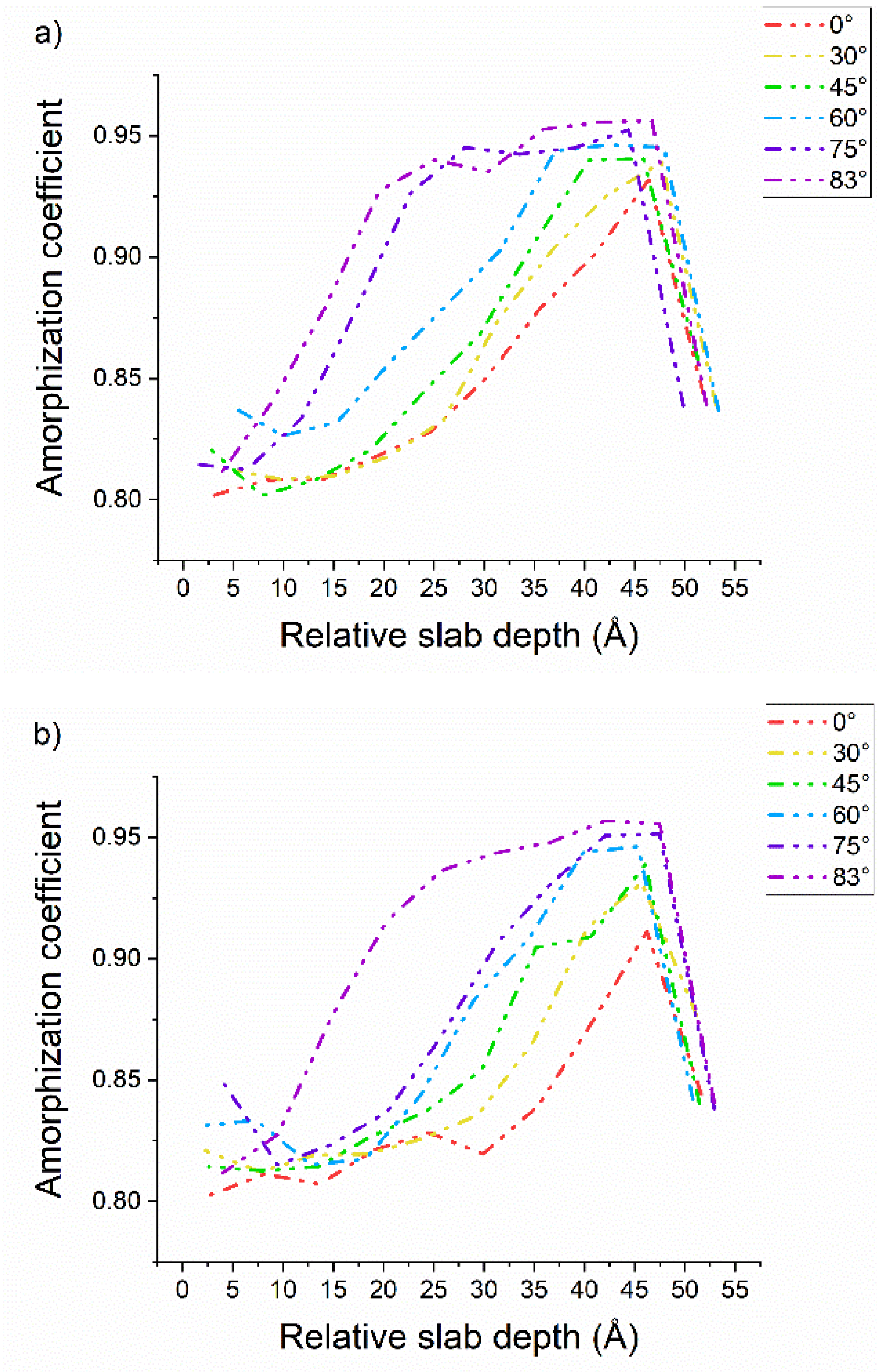


**Figure S1:** Evolution of the amorphization coefficient with respect to the incidence angle for a) 400 eV and b) 500 eV impact energies.

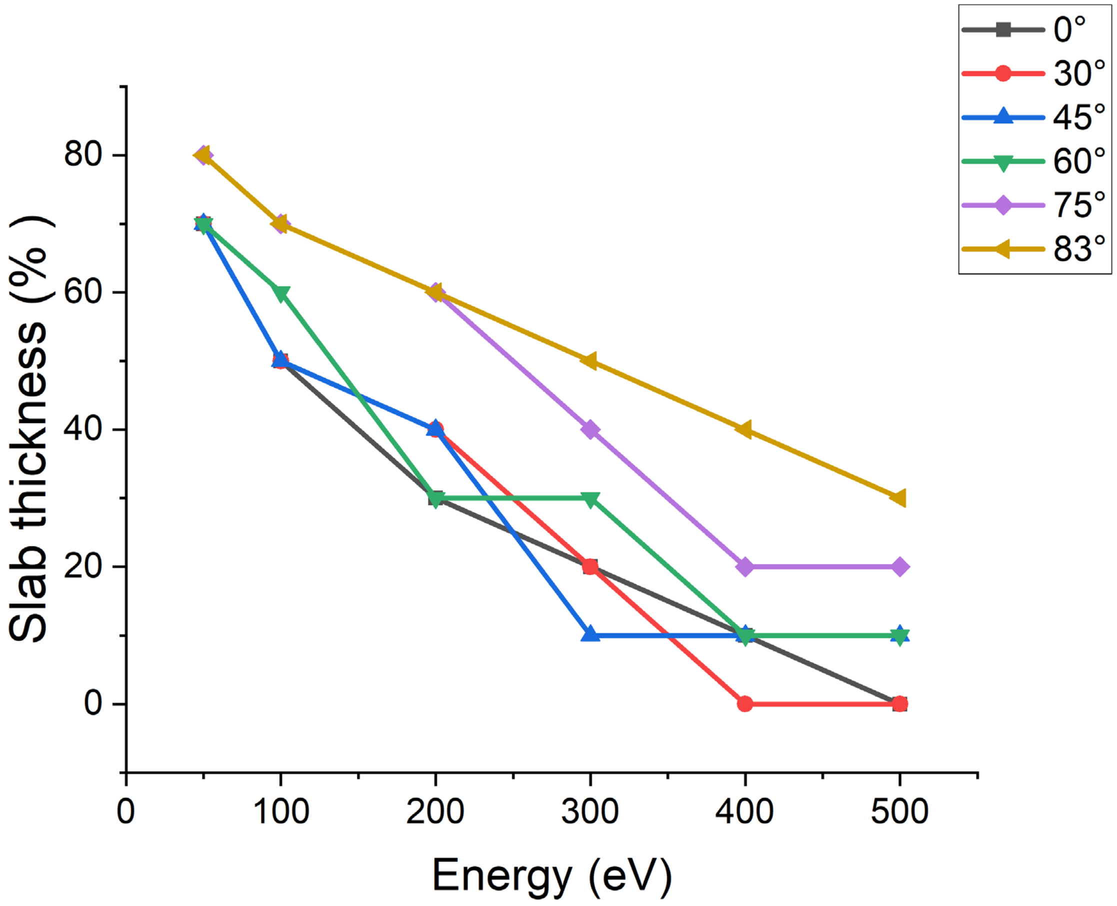


**Figure S2:** Evolution of the thickness of the crystalline slab for each energy with respect to the angle.

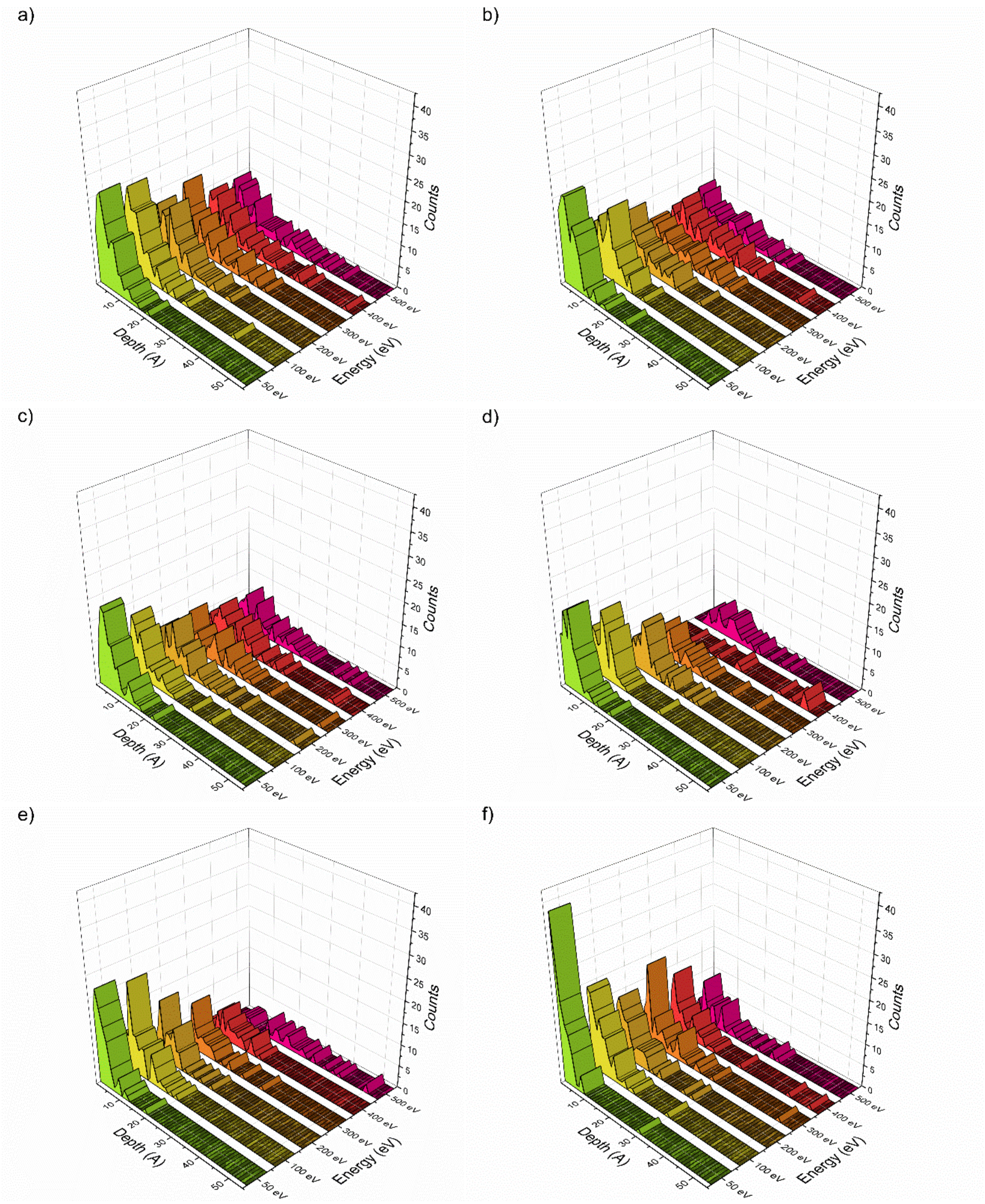


**Figure S3:** Implantation depths of oxygen for incidence angles of a) 0°, b) 30°, c) 45°, d) 60°, e) 75° and f) 83°. Each energy is displayed along the y axis, while the implantation depth is along the x and the number of counts is displayed along the z axis.

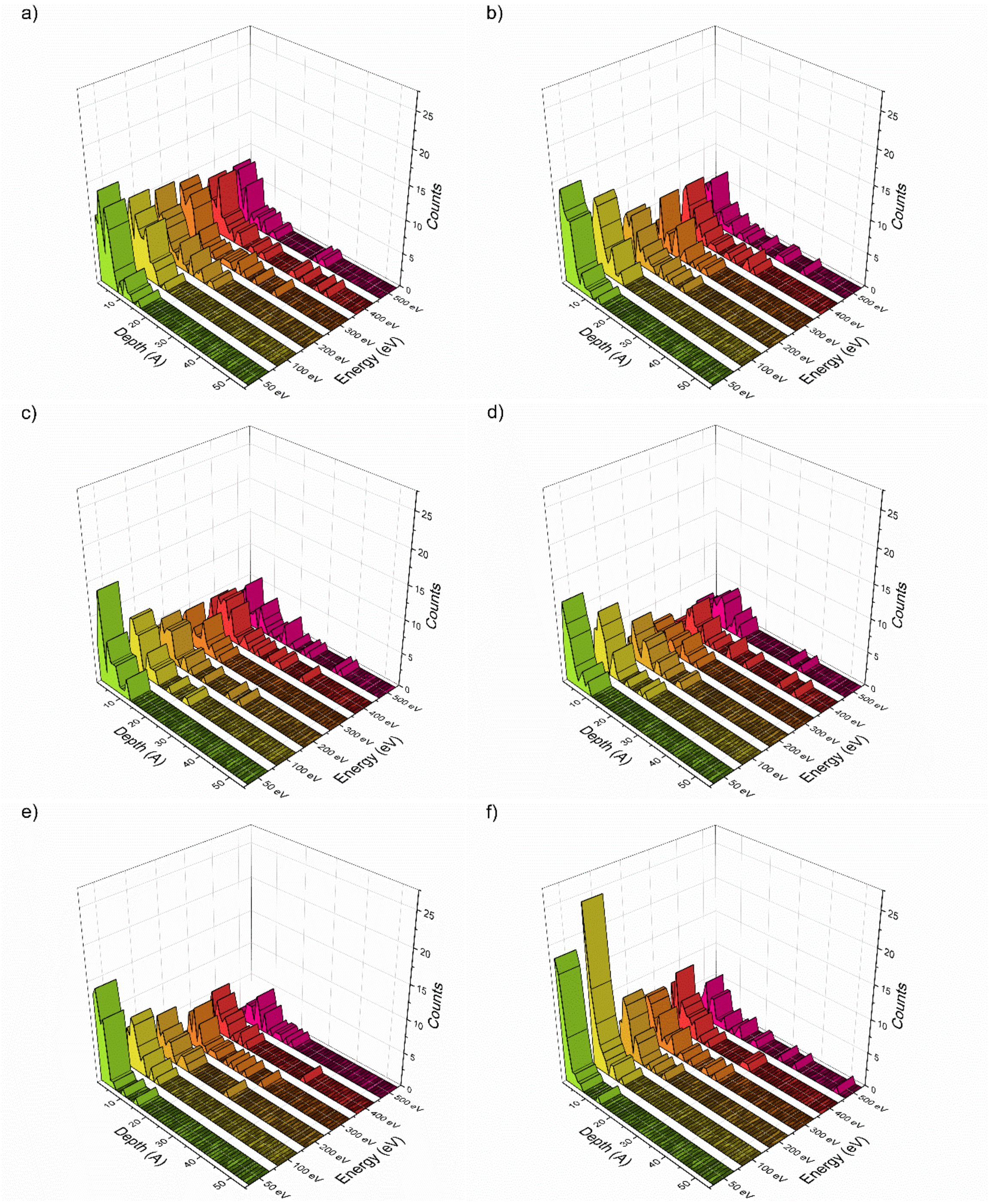


**Figure S4:** Implantation depths of hydrogen for incidence angles of a) 0°, b) 30°, c) 45°, d) 60°, e) 75° and f) 83°. Each energy is displayed along the y axis, while the implantation depth is along the x and the number of counts is displayed along the z axis.

## Sputtering of clusters

For higher impact energies, material can be sputtered in different ways:

- Direct sputtering by elastic collisions [46] from the incident argon. This means the argon collides directly with either a contaminant, a silicon or an argon atom and sputters it.
- Indirect sputtering by elastic chocs from the collision cascade. In this case, the initial collision between the incident argon and a target atom transmits the energy into the lattice, which leads possibly to other displaced atoms in the sample. These displacements can sputter particles when occurring at the sample surface.
- Backscattering of Argon particles. This mechanism usually concerns only argon that is a noble gas and therefore does not form bonds in the sample. This causes argon to be very volatile.

These mechanisms play a role in the final sputtering yields. An interesting outcome of the simulations are the mechanisms leading to the sputtering of clusters. In Figure S5 we plotted the results of the analysis of the sputtered clusters with the highest sputter yields. We included several other partial sputtering yields in the supporting information, such as oxygen – oxygen. A note on the method, clusters were detected using a threshold on interatomic distance. For silicon clusters, the interatomic threshold we set is inferior to the Si – Si bond length for clustering. Consequently, a direct observation for silicon – silicon clusters in general is that in most cases, silicon atoms are sputtered as $Si_2$ clusters. The partial Si – Si yield is also a non-negligible fraction of the pure silicon yield (up to 40% for the angles between 60 and 75°). This mechanism is enhanced for increasing impact energies and for larger incidence angles. The second most abundant sputtered clusters

are entire water molecules. Since they are deposited on the sample surface, there is at the beginning of the bombardment a high probability to sputter intact water molecules, or to fraction them. Following bombardments can also sputter silicon – oxygen clusters. Due to the strong bond between silicon and oxygen, the silicon – oxygen pair has the same sputtering yield than silicon – hydrogen, despite the fact of hydrogen being twice as abundant in the sample. Finally, very few oxygen – hydrogen, oxygen – oxygen and hydrogen – hydrogen clusters are sputtered (cf. Figure S6).

The previously described trends are increased by higher impact energies and larger incidence angles: the sputtering yield of silicon – silicon clusters is enhanced in these conditions similarly to the silicon sputtering yield. We can also observe a similar trend for water related clusters. The trend for water removal is biased by the non-renewal of the water layer, yet we can observe that higher impact energies and incidence angles favor the removal of water molecules, with a maximum at 500 eV and 75°. We can also observe good conditions of water removal in combination with a lower sputtering yield for silicon at 83°. Since the water layer is not renewed in between bombardments, another interesting observation is to measure the probability per collision to fraction a water molecule in order to determine if some water molecules remain intact on the surface after the 500 bombardments.

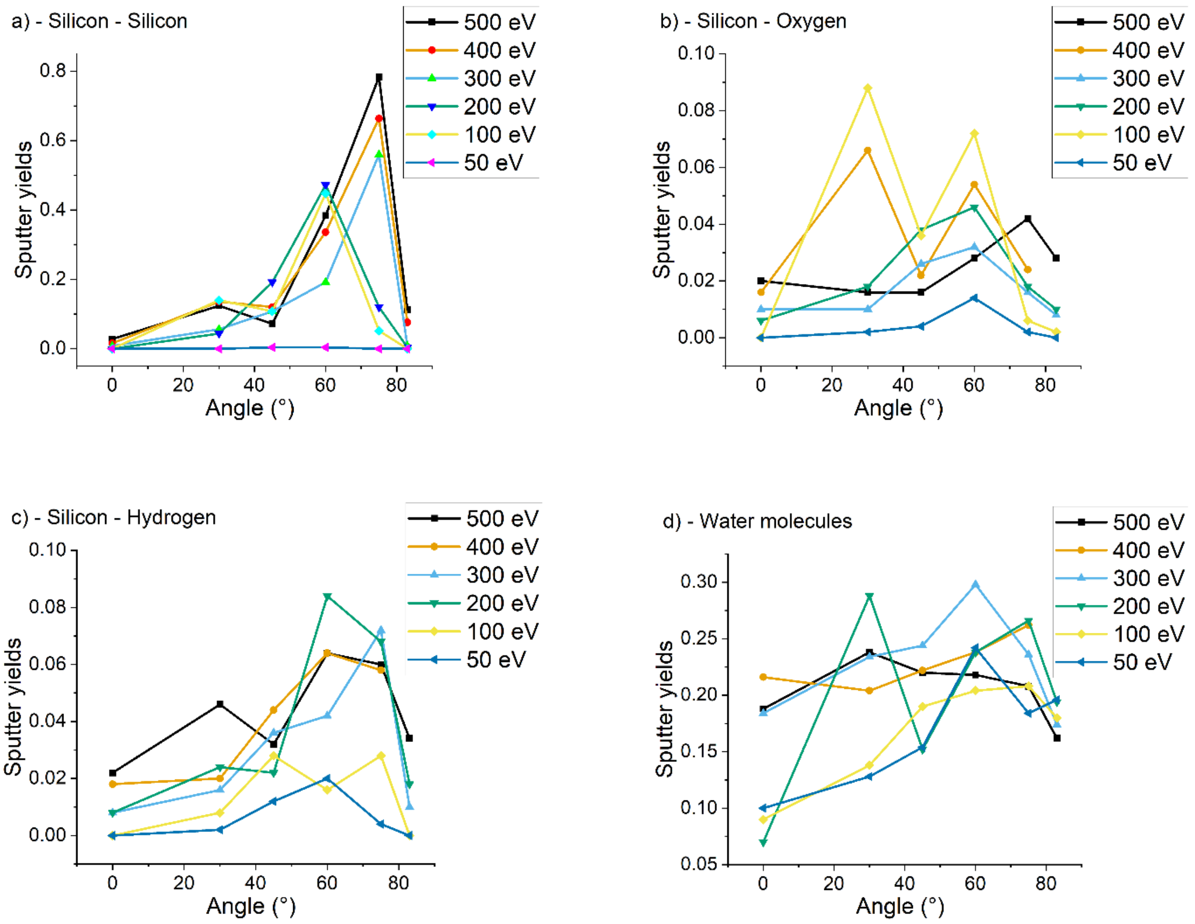


**Figure S5:** Partial sputter yields of clusters: a) silicon – silicon, b) silicon – oxygen, c) silicon – hydrogen, and d) entire water molecules.

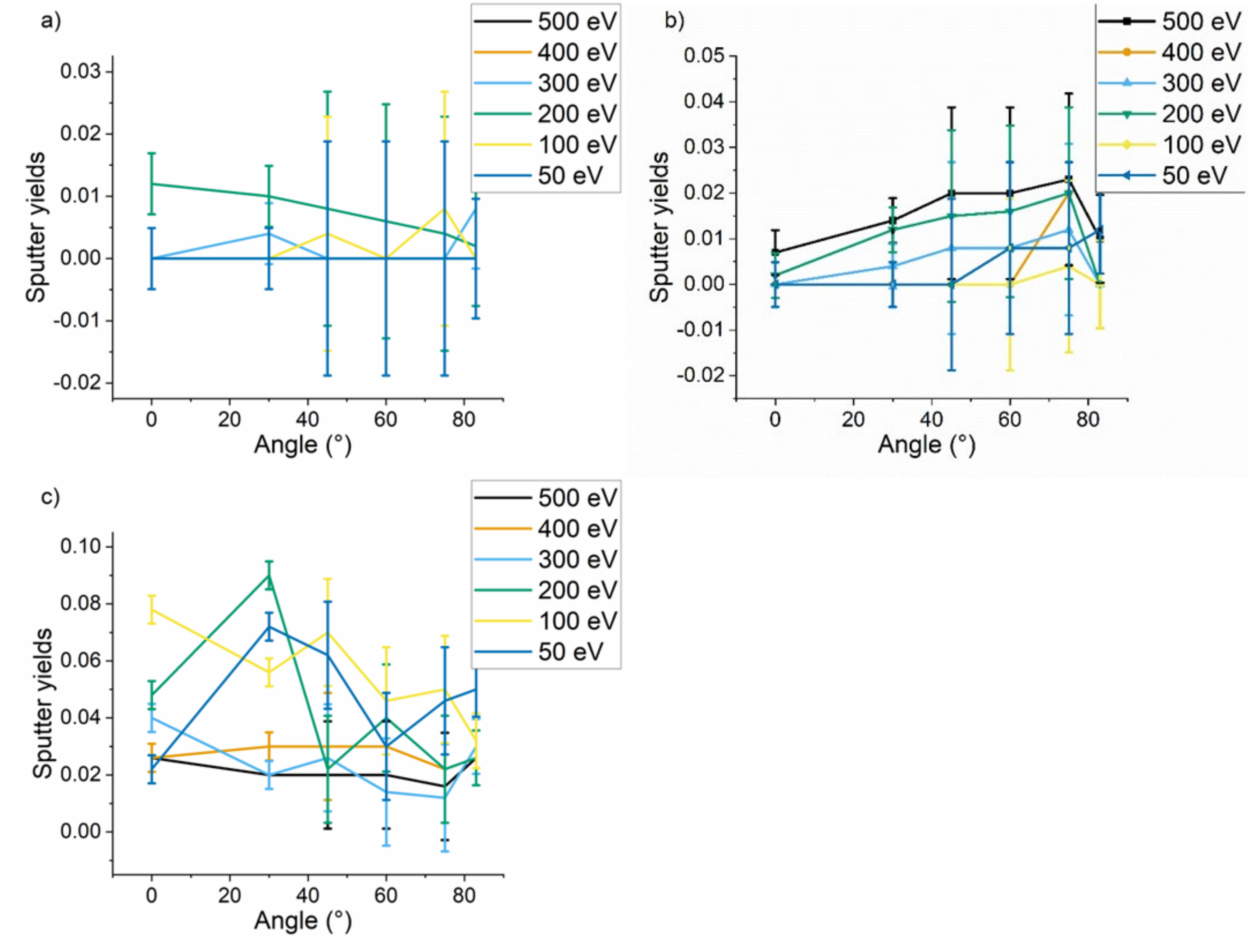


**Figure S6:** Partial sputter yields particles clusters: a) oxygen - oxygen, b) hydrogen – hydrogen and c) hydrogen – oyxgen.

From these observations we can conclude that silicon sputtering is mainly done via clusters. When comparing the silicon yield to the cluster yield, we measure that in approximatively 70% of the cases, silicon is sputtered as a $Si_2$ cluster. It is interesting to observe that a non-negligible fraction of the sputtered clusters are including a contaminating particle, approximatively 20% when adding the yield of Si – O and Si – H.

Considering the fixed amount of water molecules initial, and a substantially lower count of single hydrogen and oxygen particles in the sample, this indicates that the contaminating particles will strongly interact with the silicon particles. Furthermore, even though the atomic ratio of oxygen should be half the atomic ratio of hydrogen, the measured clusters is similar between Si – O and Si – H. This underlines the strong interaction happening between silicon and oxygen particles. Once the contaminating particles are split, they will stick to silicon and consequently will modify the local structure, allowing for these clusters to be sputtered in the later part of the bombardments.

## Evolution of the fraction of intact water molecules

The implantation of water species and the amorphization of the sample is strongly correlated to the fragmentation of water molecules during the argon bombardment. Indeed, when water molecules are dissociated, free oxygen atoms or O – H fragments are liberated near the surface, increasing the probability of the formation of a silicon – oxygen bond. Once formed, a silicon – oxygen bond will be notably hard to break and will tend to be sputtered as a whole, as shown in previous section. Thus, water fragmentation plays a key role in the amorphization of the sample, since the bond of Si – O clusters will be i) much stronger than Si – Si or Si – H, and ii) shorter than Si – Si bonds. Therefore, it is easy to understand how the formation of Si – O bonds will modify the local structure, and thus, as shown in previous sections, have an impact on the amorphization coefficient. One of the objectives, i.e. minimizing the impact of the water contamination on the sputtering process, is to remove as much water molecule as possible by sputtering, while minimizing its fragmentation and implantation. In Figure S7 the probability (in percentage)

to fragment one water molecule per impact is plotted with respect to the fluence. Since the water layer is not renewed throughout the simulations, at the end of the bombardments the number of intact water molecules tends towards zero. The evolution of the water dissociation probability can be fitted by the $Ae^{(B/x)}$ function. A steep curve indicates fast water fragmentation, while a slowly decreasing curve indicates the presence of intact water particles until high fluences. With increasing bombardment energy, we can observe an increased water fragmentation probability: a higher energy induces bigger collisions cascades, which in return have a higher probability to interact with water molecules and thus have a higher fragmentation probability. Despite this increased probability of fragmentation, we observed that even at a 50 eV impact energy, there are almost no intact water molecules left at the end of the simulations. We also observe that an incidence angle of 60° maximizes the water fragmentation for low impact energies. For high impact energies, the water dissociation probability is highest for large incidence angles. When considering the overall behavior of the water molecules, there is a competition between the water molecule fragmentation and its sputtering. We observed in the sputtering yield section that the intact water molecule yield is maximum at higher incidence angles and impact energies. The probability of fragmentation also increases with respect to the angle and energy. In Figure S8 we plotted the water dissociation probability for 50 and 500 eV collisions, for each angle we selected. From this plot we can deduce a lower chance of water fragmentation at lower energies. At an incidence angle of 83° we observe the lowest probability to fragment water molecules for both 50 and 500 eV. On the other hand, at 500 eV, we still have a significant intact water molecules

sputtering yield which could indicate an interesting set of parameters for water removal while also retaining a shallow amorphous layer on the sample surface.

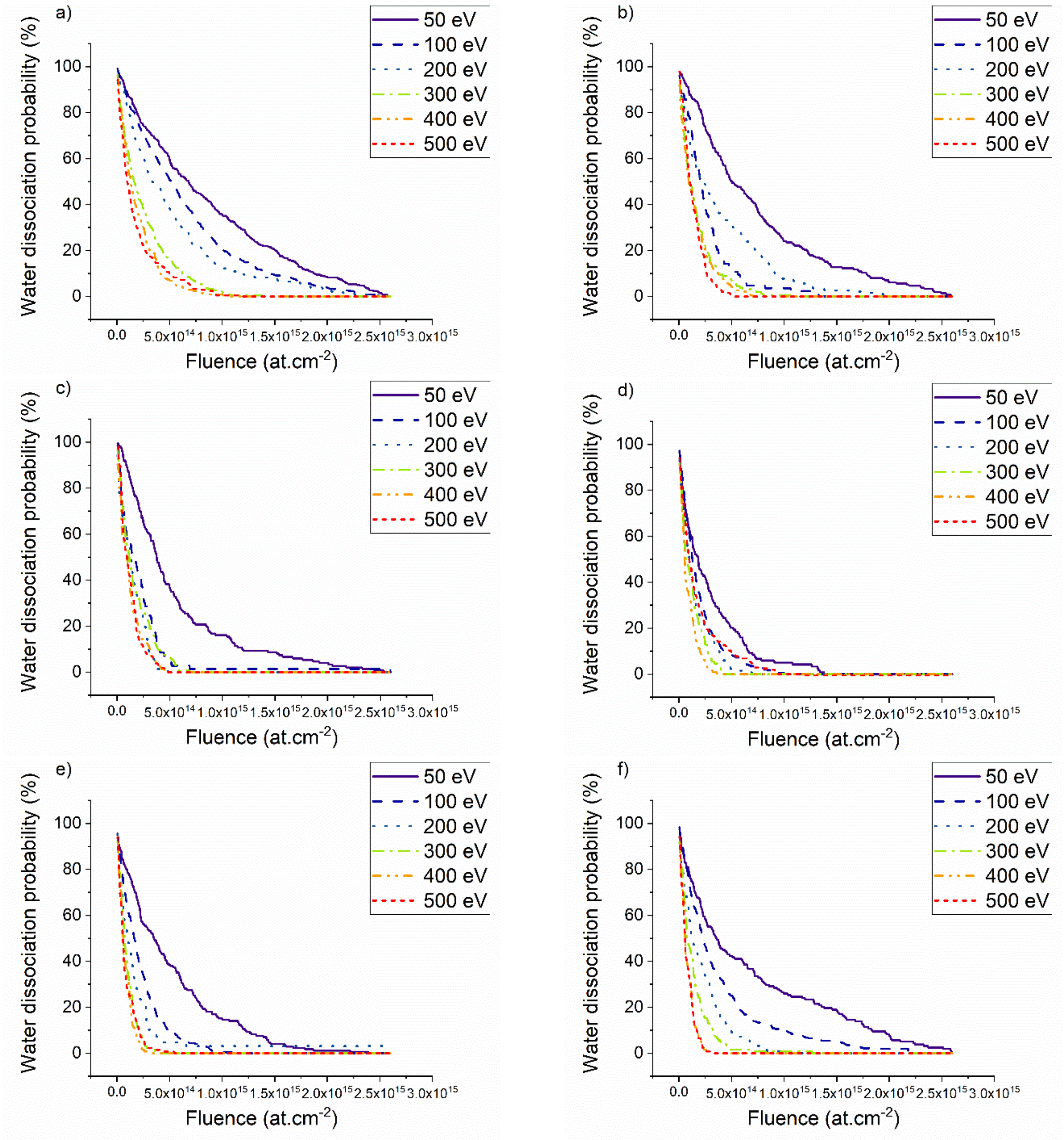


**Figure S7:** Probability to fragmentation a water molecule per impact with respect to the fluence for each energy at a) 0°, b) 30°, c) 45°, d) 60°, e) 75°, f) 83°.

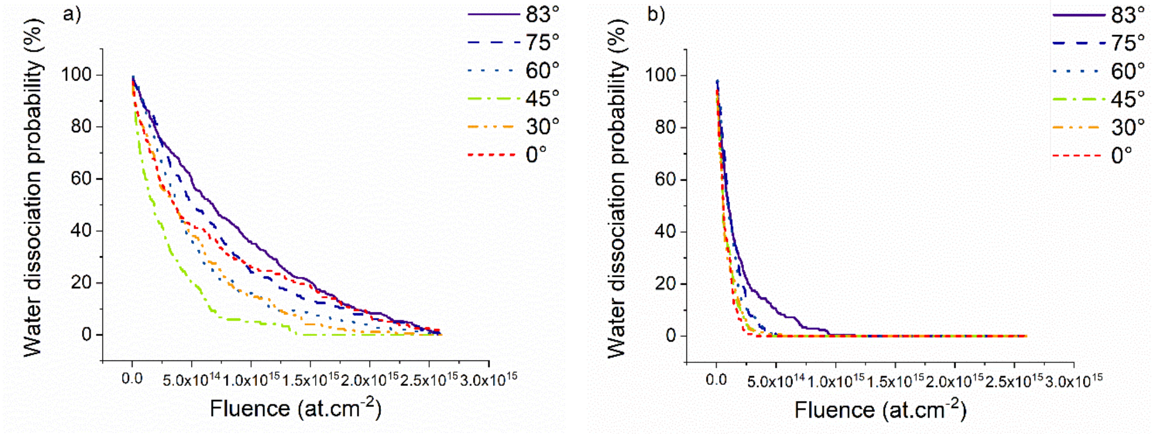


**Figure S8:** Probability to fragment a water molecule per impact with respect to the fluence for each angle for a) 50 eV impacts and b) 500 eV impacts.

When combining the information of the water fraction with the sputtered cluster, it becomes clearer how much the water contamination will impact the sputtering processes. Due to the high probability of fractioning a water molecule in the earlier stages of the irradiation process, the remaining oxygen and hydrogen particles will have a strong probability to directly interact with silicon particles. Due to this interaction, it will create scenario in which the presence of oxygen or hydrogen will enhance the sputtering of silicon. This behaviour in our simulation remains limited (up to 20% as shown in the previous paragraph), yet, in a scenario where the water layer would be renewed, this could lead to a substantial increase in the sputtering yield of silicon due to the presence of water.

## ReaxFF potential

**Table S1:** Details of the ReaxFF potential used in the simulations.

Reactive MD-force field: c/h/o/Si/C August 3

39 ! Number of general parameters

50.0000 !Overcoordination parameter

9.5469 !Overcoordination parameter

26.5405 !Valency angle conjugation parameter

1.5105 !Triple bond stabilisation parameter

6.6630 !Triple bond stabilisation parameter

0.0000 !C2-correction

1.0588 !Undercoordination parameter

4.6000 !Triple bond stabilisation parameter

12.1176 !Undercoordination parameter

13.3056 !Undercoordination parameter

-70.1292 !Triple bond stabilization energy

0.0000 !Lower Taper-radius

10.0000 !Upper Taper-radius

2.8793 !Not used

33.8667 !Valency undercoordination

6.0891 !Valency angle/lone pair parameter

1.0563 !Valency angle

2.0384 !Valency angle parameter

6.1431 !Not used

6.9290 !Double bond/angle parameter

0.3989 !Double bond/angle parameter: overcoord

3.9954 !Double bond/angle parameter: overcoord

-2.4837 !Not used

5.7796 !Torsion/BO parameter

10.0000 !Torsion overcoordination

1.9487 !Torsion overcoordination

-1.2327 !Conjugation 0 (not used)

2.1645 !Conjugation

1.5591 !vdWaals shielding

0.1000 !Cutoff for bond order (*100)

2.1365 !Valency angle conjugation parameter

0.6991 !Overcoordination parameter

50.0000 !Overcoordination parameter

1.8512 !Valency/lone pair parameter

0.5000 !Not used

20.0000 !Not used

5.0000 !Molecular energy (not used)

0.0000 !Molecular energy (not used)

2.6962 !Valency angle conjugation parameter

7 ! Nr of atoms; cov.r; valency;a.m;Rvdw;Evdw;gammaEEM;cov.r2;#

alfa;gammavdW;valency;Eunder;Eover;chiEEM;etaEEM;n.u.

cov r3;Elp;Heat inc.;n.u.;n.u.;n.u.;n.u.

ov/un;val1;n.u.;val3,vval4

C 1.3825 4.0000 12.0000 1.9133 0.1853 0.9000 1.1359 4.0000

9.7602 2.1346 4.0000 33.2433 79.5548 5.8678 7.0000 0.0000

1.2104 0.0000 199.0303 8.6991 34.7289 13.3894 0.8563 0.0000

-2.8983 2.5000 1.0564 4.0000 2.9663 0.0000 0.0000 0.0000

H 0.7853 1.0000 1.0080 1.5904 0.0419 1.0206 -0.1000 1.0000

9.3557 5.0518 1.0000 0.0000 121.1250 5.3200 7.4366 1.0000

-0.1000 0.0000 62.4879 1.9771 3.3517 0.7571 1.0698 0.0000

-15.7683 2.1488 1.0338 1.0000 2.8793 0.0000 0.0000 0.0000

O 1.2477 2.0000 15.9990 1.9236 0.0904 1.0503 1.0863 6.0000

10.2127 7.7719 4.0000 36.9573 116.0768 8.5000 8.9989 2.0000

0.9088 1.0003 60.8726 20.4140 3.3754 0.2702 0.9745 0.0000

-3.6141 2.7025 1.0493 4.0000 2.9225 0.0000 0.0000 0.0000

N 1.2333 3.0000 14.0000 1.9324 0.1376 0.8596 1.1748 5.0000

10.0667 7.8431 4.0000 32.2482 100.0000 6.8418 6.3404 2.0000

1.0433 13.7673 119.9837 2.1961 3.0696 2.7683 0.9745 0.0000

-4.3875 2.6192 1.0183 4.0000 2.8793 0.0000 0.0000 0.0000

S 1.9401 2.0000 32.0600 2.0629 0.2095 1.0316 1.5483 6.0000

9.9553 4.9055 4.0000 52.9998 112.1416 6.5181 8.2345 2.0000

1.4601 9.6977 71.1843 5.7487 23.2859 12.7147 0.9745 0.0000

-11.0200 2.7266 1.0338 6.2998 2.8793 0.0000 0.0000 0.0000

Si 2.0291 4.0000 28.0600 2.0043 0.1247 0.8218 1.5023 4.0000

13.0000 2.0618 4.0000 11.8211 136.4845 1.8038 7.3852 0.0000

-1.0000 0.0000 126.5182 3.6038 8.5961 0.2368 0.8563 0.0000

-3.5163 4.2105 1.0338 6.2998 2.5791 0.0000 0.0000 0.0000

X -0.1000 2.0000 1.0080 2.0000 0.0000 1.0000 -0.1000 6.0000

10.0000 2.5000 4.0000 0.0000 0.0000 8.5000 15.0000 0.0000

-0.1000 0.0000 127.6226 8.7410 13.3640 0.6690 0.9745 0.0000

-11.0000 2.7466 1.0338 6.2998 2.8793 0.0000 0.0000 0.0000

19 ! Nr of bonds; Edis1;LPpen;n.u.;pbe1;pbo5;13corr;pbo6

pbe2;pbo3;pbo4;n.u.;pbo1;pbo2;ovcorr

1 1 156.5953 100.0397 80.0000 -0.8157 -0.4591 1.0000 37.7369 0.4235

0.4527 -0.1000 9.2605 1.0000 -0.0750 6.8316 1.0000 0.0000

1 2 170.2316 0.0000 0.0000 -0.5931 0.0000 1.0000 6.0000 0.7140

5.2267 1.0000 0.0000 1.0000 -0.0500 6.8315 0.0000 0.0000

2 2 156.0973 0.0000 0.0000 -0.1377 0.0000 1.0000 6.0000 0.8240

2.9907 1.0000 0.0000 1.0000 -0.0593 4.8358 0.0000 0.0000

1 3 160.4802 105.1693 23.3059 -0.3873 -0.1613 1.0000 10.8851 1.0000

0.5341 -0.3174 7.0303 1.0000 -0.1463 5.2913 0.0000 0.0000

3 3 60.1463 176.6202 51.1430 -0.2802 -0.1244 1.0000 29.6439 0.9114

0.2441 -0.1239 7.6487 1.0000 -0.1302 6.2919 1.0000 0.0000

1 4 134.1215 140.2179 79.9745 0.0163 -0.1428 1.0000 27.0617 0.2000

0.1387 -0.3681 7.1611 1.0000 -0.1000 5.0825 1.0000 0.0000

3 4 130.8596 169.4551 40.0000 0.3837 -0.1639 1.0000 35.0000 0.2000

1.0000 -0.3579 7.0004 1.0000 -0.1193 6.8773 1.0000 0.0000

4 4 157.9384 82.5526 152.5336 0.4010 -0.1034 1.0000 12.4261 0.5828

0.1578 -0.1509 11.9186 1.0000 -0.0861 5.4271 1.0000 0.0000

2 3 180.4373 0.0000 0.0000 -0.8074 0.0000 1.0000 6.0000 0.5514

1.2490 1.0000 0.0000 1.0000 -0.0657 5.0451 0.0000 0.0000

2 4 231.8173 0.0000 0.0000 -0.3364 0.0000 1.0000 6.0000 0.4402

8.8910 1.0000 0.0000 1.0000 -0.0327 6.5754 0.0000 0.0000

1 5 129.1942 74.3656 55.2528 0.1066 -0.5211 1.0000 18.9617 0.5950

0.2950 -0.2398 8.0314 1.0000 -0.1019 5.6754 1.0000 0.0000

2 5 151.3159 0.0000 0.0000 -0.4644 0.0000 1.0000 6.0000 0.5950

9.4365 1.0000 0.0000 1.0000 -0.0303 7.0100 1.0000 0.0000

3 5 0.0000 0.0000 0.0000 0.5563 -0.4038 1.0000 49.5611 0.6000

0.4259 -0.4577 12.7569 1.0000 -0.1100 7.1145 1.0000 0.0000

4 5 0.0000 0.0000 0.0000 0.4438 -0.2034 1.0000 40.3399 0.6000

0.3296 -0.3153 9.1227 1.0000 -0.1805 5.6864 1.0000 0.0000

5 5 96.1871 93.7006 68.6860 0.0955 -0.4781 1.0000 17.8574 0.6000

0.2723 -0.2373 9.7875 1.0000 -0.0950 6.4757 1.0000 0.0000

1 6 90.6281 6.3660 0.0000 0.3176 -0.5558 1.0000 17.2117 0.5577

0.7223 -0.2118 7.7440 1.0000 -0.1039 5.4442 1.0000 0.0000

2 6 137.1002 0.0000 0.0000 -0.1902 0.0000 1.0000 6.0000 0.4256

17.7186 1.0000 0.0000 1.0000 -0.0377 6.4281 0.0000 0.0000

3 6 230.7615 93.6959 43.3991 -0.3617 -0.3000 1.0000 36.0000 0.3161

0.9856 -0.3882 4.6686 1.0000 -0.3960 4.5499 1.0000 0.0000

6 6 72.8867 50.0318 30.0000 0.9983 -0.3000 1.0000 16.0000 0.1000

1.0538 -0.0447 10.6176 1.0000 -0.1452 8.0404 0.0000 0.0000

11 ! Nr of off-diagonal terms; Ediss;Ro;gamma;rsigma;rpi;rpi2

1 2 0.1219 1.4000 9.8442 1.1203 -1.0000 -1.0000

2 3 0.0344 1.6800 10.3247 0.9013 -1.0000 -1.0000

2 4 0.1059 1.8290 9.7818 0.9598 -1.0000 -1.0000

1 3 0.1131 1.8523 9.8442 1.2775 1.1342 1.0621

1 4 0.1447 1.8766 9.7990 1.3436 1.1885 1.1363

3 4 0.1048 2.0003 10.1220 1.3173 1.1096 1.0206

1 5 0.1997 2.0109 9.8603 1.6611 1.3423 -1.0000

2 5 0.0938 1.8133 9.6519 1.3629 -1.0000 -1.0000

1 6 0.0250 1.7695 12.4753 1.5866 1.4409 -1.0000

2 6 0.0291 1.6805 12.5137 1.3429 -1.0000 -1.0000

3 6 0.1958 1.7958 11.1207 1.6105 1.1632 -1.0000

71 ! Nr of angles;at1;at2;at3;Thetao,o;ka;kb;pv1;pv2

1 1 1 67.2326 22.0695 1.6286 0.0000 1.7959 15.4141 1.8089

1 1 2 65.2527 14.3185 6.2977 0.0000 0.5645 0.0000 1.1530

2 1 2 70.0840 25.3540 3.4508 0.0000 0.0050 0.0000 3.0000

1 2 2 0.0000 0.0000 6.0000 0.0000 0.0000 0.0000 1.0400

1 2 1 0.0000 3.4110 7.7350 0.0000 0.0000 0.0000 1.0400

2 2 2 0.0000 27.9213 5.8635 0.0000 0.0000 0.0000 1.0400

1 1 3 49.5561 7.3771 4.9568 0.0000 0.7533 15.9906 1.0010

3 1 3 77.1171 39.8746 2.5403 -24.3902 1.7740 -42.9758 2.1240

1 1 4 66.1305 12.4661 7.0000 0.0000 3.0000 50.0000 1.1880

3 1 4 73.9544 12.4661 7.0000 0.0000 3.0000 0.0000 1.1880

4 1 4 64.1581 12.4661 7.0000 0.0000 3.0000 0.0000 1.1880

2 1 3 65.0000 14.2057 4.8649 0.0000 0.3504 0.0000 1.7185

2 1 4 74.2929 31.0883 2.6184 0.0000 0.0755 0.0000 1.0500

1 2 4 0.0000 0.0019 6.3000 0.0000 0.0000 0.0000 1.0400

1 3 1 74.3994 44.7500 0.7982 0.0000 3.0000 0.0000 1.0528

1 3 3 77.9854 36.6201 2.0201 0.0000 0.7434 67.0264 3.0000

1 3 4 82.4890 31.4554 0.9953 0.0000 1.6310 0.0000 1.0783

3 3 3 80.7324 30.4554 0.9953 0.0000 1.6310 50.0000 1.0783

3 3 4 84.3637 31.4554 0.9953 0.0000 1.6310 0.0000 1.0783

4 3 4 89.7071 31.4554 0.9953 0.0000 1.6310 0.0000 1.1519

1 3 2 71.5018 21.7062 0.4735 0.0000 0.5186 0.0000 1.1793

2 3 3 84.9468 23.3540 1.5057 0.0000 2.6374 0.0000 1.3023

2 3 4 75.6201 18.7919 0.9833 0.0000 0.1218 0.0000 1.0500

2 3 2 77.0645 10.4737 1.2895 0.0000 0.9924 0.0000 1.1043

1 4 1 66.0330 22.0295 1.4442 0.0000 1.6777 0.0000 1.0500

1 4 3 103.3204 33.0381 0.5787 0.0000 1.6777 0.0000 1.0500

1 4 4 104.1335 8.6043 1.6495 0.0000 1.6777 0.0000 1.0500

3 4 3 74.1978 42.1786 1.7845 -18.0069 1.6777 0.0000 1.0500

3 4 4 74.8600 43.7354 1.1572 -0.9193 1.6777 0.0000 1.0500

4 4 4 75.0538 14.8267 5.2794 0.0000 1.6777 0.0000 1.0500

1 4 2 69.1106 25.5067 1.1003 0.0000 0.0222 0.0000 1.0369

2 4 3 81.3686 40.0712 2.2396 0.0000 0.0222 0.0000 1.0369

2 4 4 83.0104 43.4766 1.5328 0.0000 0.0222 0.0000 1.0500

2 4 2 70.8687 12.0168 5.0132 0.0000 0.0222 0.0000 1.1243

1 2 3 0.0000 25.0000 3.0000 0.0000 1.0000 0.0000 1.0400

1 2 4 0.0000 0.0019 6.0000 0.0000 0.0000 0.0000 1.0400

1 2 5 0.0000 0.0019 6.0000 0.0000 0.0000 0.0000 1.0400

3 2 3 0.0000 0.0148 6.0000 0.0000 0.0000 0.0000 1.0400

3 2 4 0.0000 0.0019 6.0000 0.0000 0.0000 0.0000 1.0400

4 2 4 0.0000 0.0019 6.0000 0.0000 0.0000 0.0000 1.0400

2 2 3 0.0000 9.7025 6.0000 0.0000 0.0000 0.0000 1.0400

2 2 4 0.0000 0.0019 6.0000 0.0000 0.0000 0.0000 1.0400

1 1 5 73.9923 24.7559 1.8287 0.1463 0.0059 0.0000 1.0600

1 5 1 86.7521 36.5756 2.0199 0.1463 0.0058 0.0000 1.0600

2 1 5 75.1310 24.8619 1.8104 0.0000 0.0050 0.0000 1.0600

1 5 2 85.3326 36.9451 2.1403 0.0000 0.0388 0.0000 1.0706

1 5 5 86.0081 37.0451 2.1403 0.1463 0.1070 0.0000 1.0098

2 5 2 92.9959 36.9602 2.0403 0.0000 0.0050 0.0000 1.0200

2 5 5 83.2918 36.9451 2.0199 0.0000 0.0050 0.0000 1.0600

2 2 5 0.0000 0.0019 6.0000 0.0000 0.0000 0.0000 1.0400

6 6 6 71.6771 13.0081 3.6376 0.0000 0.2384 0.0000 1.3185

2 6 6 89.1207 11.7566 1.1579 0.0000 0.0100 0.0000 1.2975

2 6 2 26.3763 5.5393 0.9656 0.0000 2.3381 0.0000 1.1704

3 6 6 85.6335 17.1826 6.5759 0.0000 0.4105 0.0000 1.6398

2 6 3 59.6558 6.8748 7.0452 0.0000 4.0000 0.0000 1.0400

3 6 3 72.7359 17.5203 2.4434 0.0000 0.0100 0.0000 1.7374

6 3 6 18.3653 5.7702 3.4915 0.0000 4.0000 0.0000 1.9438

2 3 6 57.5894 40.0000 8.0000 0.0000 3.8263 0.0000 1.0534

3 3 6 54.5893 38.8349 7.6245 0.0000 2.7656 0.0000 3.0000

2 2 6 0.0000 47.1300 6.0000 0.0000 1.6371 0.0000 1.0400

6 2 6 0.0000 31.5209 6.0000 0.0000 1.6371 0.0000 1.0400

3 2 6 0.0000 31.0427 4.5625 0.0000 1.6371 0.0000 1.0400

1 1 6 63.8858 35.1811 0.6236 0.0000 2.6344 0.0000 2.3890

1 6 1 71.6429 31.1160 0.5107 0.0000 0.0100 0.0000 1.9113

6 1 6 63.2523 33.3810 2.2952 0.0000 0.0201 0.0000 1.7191

1 6 6 70.9876 29.7098 1.0210 0.0000 0.0100 0.0000 1.8242

2 1 6 96.9319 10.9008 1.4627 0.0000 2.4557 0.0000 1.5109

1 6 2 73.9320 16.6559 3.0433 0.0000 0.7961 0.0000 1.4005

1 3 6 91.5678 5.9243 2.4284 0.0000 2.9840 0.0000 1.0400

1 6 3 96.3796 36.5757 0.8505 0.0000 3.6964 0.0000 1.6527

3 1 6 42.5553 40.0000 1.5855 0.0000 1.0802 0.0000 1.1584

34 ! Nr of torsions;at1;at2;at3;at4;;V1;V2;V3;V2(BO);vconj;n.u;n

1 1 1 1 -0.2500 11.5822 0.1879 -4.7057 -2.2047 0.0000 0.0000

1 1 1 2 -0.2500 31.2596 0.1709 -4.6391 -1.9002 0.0000 0.0000

2 1 1 2 -0.1770 30.0252 0.4340 -5.0019 -2.0697 0.0000 0.0000

1 1 1 3 -0.7098 22.2951 0.0060 -2.5000 -2.1688 0.0000 0.0000

2 1 1 3 -0.3568 22.6472 0.6045 -4.0088 -1.0000 0.0000 0.0000

3 1 1 3 -0.0528 6.8150 0.7498 -5.0913 -1.0000 0.0000 0.0000

1 1 3 1 2.0007 25.5641 -0.0608 -2.6456 -1.1766 0.0000 0.0000

1 1 3 2 -1.1953 42.1545 -1.0000 -8.0821 -1.0000 0.0000 0.0000

2 1 3 1 -0.9284 34.3952 0.7285 -2.5440 -2.4641 0.0000 0.0000

2 1 3 2 -2.5000 79.6980 1.0000 -3.5697 -2.7501 0.0000 0.0000

1 1 3 3 -0.0179 5.0603 -0.1894 -2.5000 -2.0399 0.0000 0.0000

2 1 3 3 -0.5583 80.0000 1.0000 -4.4000 -3.0000 0.0000 0.0000

3 1 3 1 -2.5000 76.0427 -0.0141 -3.7586 -2.9000 0.0000 0.0000

3 1 3 2 0.0345 78.9586 -0.6810 -4.1777 -3.0000 0.0000 0.0000

3 1 3 3 -2.5000 66.3525 0.3986 -3.0293 -3.0000 0.0000 0.0000

1 3 3 1 2.5000 -0.5332 1.0000 -3.5096 -2.9000 0.0000 0.0000

1 3 3 2 -2.5000 3.3219 0.7180 -5.2021 -2.9330 0.0000 0.0000

2 3 3 2 2.2500 -6.2288 1.0000 -2.6189 -1.0000 0.0000 0.0000

1 3 3 3 0.0531 -17.3983 1.0000 -2.5000 -2.1584 0.0000 0.0000

2 3 3 3 0.4723 -12.4144 -1.0000 -2.5000 -1.0000 0.0000 0.0000

3 3 3 3 -2.5000 -25.0000 1.0000 -2.5000 -1.0000 0.0000 0.0000

0 1 2 0 0.0000 0.0000 0.0000 0.0000 0.0000 0.0000 0.0000

0 2 2 0 0.0000 0.0000 0.0000 0.0000 0.0000 0.0000 0.0000

0 2 3 0 0.0000 0.1000 0.0200 -2.5415 0.0000 0.0000 0.0000

0 1 1 0 0.0000 50.0000 0.3000 -4.0000 -2.0000 0.0000 0.0000

0 3 3 0 0.5511 25.4150 1.1330 -5.1903 -1.0000 0.0000 0.0000

0 1 4 0 -2.4242 128.1636 0.3739 -6.6098 -2.0000 0.0000 0.0000

0 2 4 0 0.0000 0.1000 0.0200 -2.5415 0.0000 0.0000 0.0000

0 3 4 0 1.4816 55.6641 0.0004 -7.0465 -2.7831 0.0000 0.0000

0 4 4 0 -0.3244 27.7086 0.0039 -2.8272 -2.0000 0.0000 0.0000

4 1 4 4 -5.5181 8.9706 0.0004 -6.1782 -2.0000 0.0000 0.0000

0 1 5 0 0.1515 29.0501 0.0792 -4.5064 -1.0200 0.0000 0.0000

0 5 5 0 -0.0054 0.1000 0.1715 -2.2256 -1.0000 0.0000 0.0000

0 2 5 0 0.0000 0.0000 0.0000 0.0000 0.0000 0.0000 0.0000

0 ! Nr of hydrogen bonds;at1;at2;at3;Rhb;Dehb;vhb1

3 2 3 1.9682 -4.4628 1.7976 3.0000

3 2 4 2.0000 -6.0000 1.7976 3.0000

4 2 3 1.2000 -2.0000 1.7976 3.0000

4 2 4 1.2979 -6.0000 1.7976 3.0000

3 2 5 1.5000 -2.0000 1.7976 3.0000

4 2 5 1.5000 -2.0000 1.7976 3.0000

5 2 3 1.5000 -2.0000 1.7976 3.0000

5 2 4 1.5000 -2.0000 1.7976 3.0000

5 2 5 1.5000 -2.0000 1.7976 3.0000
